# European Musculo-Skeletal Oncology Society Meeting, 4 & 5 May 2000, London: Abstracts and Poster Presentations and Nurses Symposium

**DOI:** 10.1080/135771401200X8629

**Published:** 2001-06


*Sarcoma* (2001) **5**, 105–127

### European Musculo-Skeletal Oncology Society Meeting, 4 & 5 May 2000, London: Abstracts and Poster Presentations and Nurses Symposium

### Oral presentations

### Prognostic factors predicting survival in patients with high-grade osteosarcoma located in the extremity or trunk

S. Bielack, B. Kempf-Bielack, G. Delling, U. Heise, K. Helmke, T. Huf, H. Jürgens, M. Kevric, R. Kotz, S. Lang & A. Zoubek, for the Cooperative OsteoSarcoma Study Group COSS
*(Dept. of Ped. Hematology/Oncology, University Muenster, Germany)*

*Introduction:* An analysis was performed in order to determine which of the prospectively collected variables included in the COSS-database might correlate with survival in osteosarcoma.
*Patients and methods:* All 1702 patients with previously untreated (except primary surgery) high-grade osteosarcoma of the extremity or trunk registered into a neoadjuvant COSS-study before 7/98 were included. Factors analyzed by Kaplan–Meier analysis and Cox-regression were patient age (1648 <; 54 x ≥ 40 years) and sex (1007 male; 695 female), tumor site (1595 limb; 107 axial; for limbs: 240 proximal; 1355 distal), tumor size (1037 <; 523 ≥ 1/3 of involved bone, data available for extremity tumors only), primary metastases (1491 no; 211 yes), osteosarcoma as first (1670) or secondary (32) malignancy, lag-time before diagnostic biopsy (576 <; 560 ≥ 70 days), time from biopsy to therapy (1524 ≤ 178 > 3 weeks), timing of surgery (157 primary; 1451 delayed), type of surgery (881 ablative; 720 limb-salvage), response to preoperative chemotherapy (734 good (<10% viable tumor); 586 poor), and best surgical remission (1510 macroscopically complete; 180 incomplete).
*Results:* At last-follow-up, 1160 of 1702 patients were alive (median follow-up 5.5 years for survivors; range, 9 days–19.0 years). Actuarial 5, 10, and 15 year survival rates were 65.3%, 59.8%, and 57.3%, respectively. In univariate analyses, age (actuarial survival: young 57.7%, old 41.6%), site (appendicular 59.0%, axial 29.3%; for extremity tumors: distal 61.2%, proximal 47.1%), size (small 64.7%, large 48.6%), primary metastases (no 61.6%, yes 26.7%), response (good 72.6%, poor 43.1%), and surgical remission (yes 60.1%, no 10.9%) were significant prognostic factors. All factors but age kept their significance in multivariate testing, with surgical remission, response and primary metastases showing the highest odds ratios. Except for a few irradiated patients, long-term survival without complete surgery of all lesions was almost exclusively limited to patients with presumed primary metastases which lacked histological proof.
*Conclusion:* Primary metastases, tumor site and size are prognostic factors which can be used to risk-stratify osteosarcoma patients at diagnosis. A realistic assessment of an individual patient's prognosis, however, can only be made later in the course of treatment, when information on surgical remission and response to chemotherapy can be included into the calculation.
*Supported by Deutsche Krebshilfe*

### Prolonged follow-up of extremity osteosarcoma. Results from the European Osteosarcoma Intergroup

J.S. Whelan, S. Weeden, B. Uscinska & A. McTiernan for the European Osteosarcoma Intergroup (EOI)

*Study aims:* To describe the results of two large randomised trials of neo-adjuvant chemotherapy for extremity osteosarcoma (OS) after prolonged follow up.
*Background:* Reported survival rates for extremity OS range from 50 to over 70%. The best results have been reported from single centres or from non-randomised studies but these results are potentially subject to significant bias. To minimise such bias, the EOI has conducted large multicentre randomised studies of chemotherapy for OS. The first two studies have been analysed with 94% of the participants having been followed to death or beyond 5 years.
*Methods:* In the first study patients were randomised to receive the combination of doxorubicin and cisplatin with or without the addition of high-dose methotrexate. The second study compared doxorubicin and cisplatin to a multiagent regimen given for 42 weeks based on the T10 programme.
*Subjects:* One hundred and seventy-nine patients were treated in the first study with either the two- or three-drug combination. In the second study, 391 patients were randomised. The median age was 16 (range 3-40). Sites of disease were femur, 315 (55%), tibia/fibula, 177 (31%) and upper limb, 78 (14%). Three hundred and seventy-three patients underwent limb salvage surgery. Data available from 332 patients showed > 90% histological necrosis in 30%. Median follow-up of these 570 patients is 9.6 years.
*Results:* Overall 5-year survival was 56% (95% CI 52–60%) and progression-free survival was 44%. Those with tibia/fibula tumours, treated by limb salvage, or with good histological response had improved survival. There were no differences based on trial, treatment, age or sex. First recurrence was local in 4%, lung only in 33%, bone in 4% and a combination in 10%. First recurrence after 5 years occurred in four patients (3%).
*Conclusion:* These data, from large multicentre trials, provide the firmest evidence of outcome for extremity OS. Future randomised studies must aim to improve survival towards that reported in open studies.

### Low grade central osteosarcoma—fibrous dysplasia and parosteal subtype: a study of 28 cases of the Hamburg Bone Tumor Registry

C. Engels, M. Werner & G. Delling
*(Abteilung Osteopathologie/Pathologisches Institut/Universität Hamburg)*

*Aim:* The central osteosarcoma is regarded as a high grade malignant tumor, whereas the low grade central osteosarcoma (LGCO) appears to be a distinct member of this group which is characterized by a more favorable clinical outcome than

1357–714X print/1369–1643 online/01/020105–23 © 2001 Taylor & Francis Ltd
DOI: 10.1080/135771401200X8629

conventional osteosarcoma. LGCOs represent less than 2% of all osteosarcomas. Morphologically it is simulating benign bone tumors (fibrous dysplasia, nonossifying fibroma, desmoplastic fibroma, chondromyxoid fibroma), tumor-like lesions (aneursymal bone cyst) or parosteal osteosarcoma. Only a small number of this rare tumor has been described so far. Clinical data and radiographic and histological features of 28 LGCOs were studied.
*Methods:* We have investigated 28 cases of LGCO (18 parosteal and 10 fibrous dysplasia-like subtype) regarding their age distribution, localization, radiological and histological features, in some cases their clinical course.
*Results:* The age distribution was broad (range 8–65 years). The primary symptom was pain, mean duration lasting 6 months. The median follow up after surgery spanning 46 months. Most cases of the parosteal subtype were treated by wide resection ($n$=11). Only one case recurred 6 months after marginal resection and developed lung metastases 15 months later. Within fibrous dysplasia subtype 4, recurrences could be identified after three intralesional curretage and one wide resection. Another case manifested three additional local recurrences and developed three consecutive lung metastases with only minor transformation into a higher grade tumor. At present no patient has died of his disease. Radiologically the parosteal subtype was more sclerotic and mainly localized in the distal femur. The fibrous dysplasia subtype was more lytic and was found in different sites of the skeleton. Despite a morphological pattern of fibrous dysplasia or parosteal osteosarcoma they histologically show spindle-shaped cells with minimal hyperchromatic and irregular nuclei. In 14 cases (50%) benign tumor or high grade malignancy was supposed by the consultant pathologist.
*Conclusions:* The knowledge of different subtypes of low grade central osteosarcoma supports and substantiates a correct diagnosis and enables a good clinical outcome by surgery alone if at least wide margins are used.
*Supported by the Deutsche Krebshilfe*

**Osteosarcoma over the age of 40**

R.J. Grimer on behalf of the European Musculoskeletal Oncology Society
*(The Royal Orthopaedic Hospital Oncology Service, Bristol Road South, Birmingham, UK)*

*Purpose:* To ascertain whether patients over the age of 40 have a different disease pattern and prognosis to younger patients with osteosarcoma.
*Background:* Osteosarcoma is principally a disease of adolescents but 13% of all patients with osteosarcoma will be over the age of 40—and to date have been excluded from most national trials of treatment of osteosarcoma. A collaborative study from EMSOS has collected data on these patients.
*Material:* Data on 486 patients have been contributed from 13 centres. The ages ranged from 40 to 89 with a mean of 54. Two hundred and seventy-five were male and 211 female. The most common sites were femur followed by pelvis and tibia and 84 had a secondary osteosarcoma—42 related to Paget's disease and 42 secondary to radiation. Thirty patients had 'low grade' osteosarcoma, the remainder having high grade tumours of which 57 had metastases at presentation. Almost half the patients had chemotherapy Surgery consisted of amputation in 118 and limb salvage surgery in 252, with 113 having endoprostheses and 15 having allografts. One hundred and eighty-four were felt to have had adequate surgical margins and 102 had close or inadeqaute margins. Eighty-five patients had radiotherapy of which 41 were palliative.
*Results:* The overall survival rates were 37% at 5 years falling to 27% at 10 years. The 5- and 10-year survival rates for stages 1, 2 and 3 tumours were, respectively: 78 and 62%; 41 and 28%; 9 and 9%. Patients over the age of 60 and patients who did not have adequate treatment not surprisingly fared worse, as did all patients with secondary osteosarcoma. The median survival time for patients with Paget's osteosarcoma was 7 months and for radiation sarcoma was 12 months. For patients with stage 2 tumours the effect of age was such that the 5-year survival rates were 45% for those less than 60 and 28% for those over 60. There was little information about effectiveness of chemotherapy but there was a trend for those with a good response to fare better than those without. Local recurrence arose in 7% of those having amputations and 22% of those having limb salvage surgery. It was closely related to margins of excision, with 27% of those having inadequate margins developing local recurrence compared to 12% of those with adequate margins.
*Conclusion:* Osteosarcoma over the age of 40 requires just as much care and multidisciplinary working as osteosarcoma in the younger age group. Patients with secondary osteosarcoma have a dismal outlook. Chemotherapy and effective surgery remain the mainstay of treatment.

**Local relapses in osteosarcoma patients: the experience of the French Society Of Pediatric Oncology (SFOP)**

E. Mascard[1], P. Violas[2], R. Kohler[2], J. Dubousset[1], N. Dupouy[3], L. Brugières[3], C. Rodary[3] & C. Kalifa[3]
*([1]Hopital St Vincent de Paul, Paris, [2]Hôpital Edouard Herriot, Lyon, [3]Institut Gustave Roussy, Villejuif, France)*

*Study aim:* To study the incidence and the outcome of local relapses in children treated for an osteosarcoma
*Methods:* We reviewed data of children registered in the SFOP OS97 prospective study for a newly diagnosed osteosarcoma. Patients were treated with pre-operative chemotherapy combining high-dose methotrexate (HDMTX) 8–12 g/m$^2$ and adriamycin. After surgery, good responders (GR) (less than 10% viable cells) received additional HDMTX and adriamycin, and bad responders a combination of ifosfamide, cis-platinum and vindesine.
*Subjects:* Two hundred and one patients were included in the study from 1987 and 1994: there were 104 good responders and 95 bad responders; 166 patients underwent a conservative surgery.
*Results:* A local relapse occurred in 16 patients (10% of the conservative surgery): 5 GR and 11 BR. All these local relapses were the first site of relapse and occurred with a median delay from diagnosis of 18 months (6–42 months). Nine of 16 patients are alive with no evidence of disease (including four patients who subsequently developed lung metastasis) with a median follow-up of 41 months after relapse. Seven patients died (median delay: 12 months).
*Conclusion:* The occurrence of a local relapse after conservative treatment of an osteosarcoma is not always fatal. A second remission and a long-term survival can be achieved by immediate amputation and appropriate second line chemotherapy. We are currently conducting the same study for the patients included in the on-going study of the SFOP (OS94 study). One hundred and ninety-seven have been included so far and the incidence and outcome of local relapse in these patients will be presented.

**Local recurrence of osteosarcoma: survival rate and management**

H. Katagiri, S.R. Cannon, T.W.R. Briggs, J. Cobb & J. Witt
*(London Bone Tumour Service, Royal National Orthopaedic Hospital, Middlesex, UK)*

*Purpose:* To assess the clinical features, development of metastases and survival rate of patients with local recurrence after the resection of osteosarcoma in a large series.
*Patients and methods:* Five hundred and thirty patients with high-grade osteosarcomas were treated between 1989 and 1998. Fifty-four patients (10%) developed local recurrence after resection and adjuvant chemotherapy. There were 38 men and 16 women with a mean age of 19 years (range 6–50). The mean follow-up was 39 months (range 7–120 months). The vast majority had clear resection margins. Histological response was category 1 in 27% of the patients, and category 2 in 73%. Clinical features, treatment, and prognosis were analysed. Survival rates were examined using Kaplan–Meier Analysis.

*Results:* The average interval between the first resection and local recurrence was 15 months (range 2–109 months). Forty-one patients (76%) had local recurrence in deep soft tissue, seven in bone, and six in subcutaneous tissue. Twenty-six patients (49%) had lung metastasis at the time of local recurrence, whilst 21 patients (38%) developed it later. Thirty patients (57%) were treated with resection of the recurrent lesion and 18 (32%) were treated with amputation. One-, 3- and 5-year survival rates after local recurrence were 0.57, 0.38, and 0.22, respectively.

*Conclusion:* (1) 87% of patients with local recurrence developed metastases either concurrently or at a later date. Immediate amputation did achieve local tumour control. However, the survival rate was not statistically higher; (2) 87% of the local recurrence arise in soft tissue. Therefore, careful attention should be paid to secure the wide margin around biopsy tract, muscle insertion to the affected bone, and neurovascular bundle at the time of initial resection.

**Prognosis after recurrence of osteosarcoma. A COSS report on 547 patients with relapse following complete remission**

B. Kempf-Bielack[1], D. Branscheid, S. Flege, M. Kevric, R. Maas, H. Jürgens, & S. Bielack for the Cooperative OsteoSarcoma Study Group COSS.

*([1]Department of Pediatric Hematology/Oncology, University Muenster, Germany)*

*Introduction:* The COSS-database was used to evaluate the prognosis after osteosarcoma relapse and to determine whether prognostic factors influencing survival post-relapse might be identified.

*Patients and methods:* Among all patients with previously untreated high-grade osteosarcoma of the extremity or trunk registered into a neoadjuvant COSS-study before 7/98, 547 patients relapsed after having previously achieved a complete surgical remission. These were evaluated for patient- and tumor-related characteristics identifiable at time of relapse which might influence post-relapse survival.

*Results:* The median interval from diagnosis of osteosarcoma to relapse was short at 1.5 (0.1–10.3) years. One hundred and nineteen relapses were detected in the first, 242 in the second, 108 in the third, 36 in the fourth, 18 in the fifth year, and only 24 later. Median follow-up post-relapse was 1.0 year for all patients and 1.8 (0–17.3) years for 174 survivors. Three hundred and seventy-three patients died, 359 of osteosarcoma, 11 of other and three of unknown causes. Actuarial survival at 2, 5, and 10 years post-relapse was 34.4, 20.6, and 15.1%. Survival post-relapse did not correlate significantly with patient age or sex, the former site or size of the primary tumor, whether primary metastases had been present, or with tumor response to first-line chemotherapy. The probability to survive increased with increasing time between diagnosis and relapse (5-year survival 9.2% if interval < 1.5 years; 32.1% if ≥ 1.5 years). Five-year survival was 30.8% for 34 patients with isolated local recurrences and 21.3% for 481 patients with metastases only, but only 3.5% for 32 patients with combined local and systemic recurrences. Metastases involved the lung in 444 (355 as only site of relapse: 5-year survival 24.3%), distant bones in 88 (44 bone only: 23.6%) and other sites in 54 patients (11 other sites only: 20.0%). Five-year survival was dismal at 4.8% for patients with involvement of more than one system (local/lung/bone/other), compared to 24.5% if only one system was involved.

*Conclusion:* Survival following osteosarcoma relapse remains poor. Risk factors pointing to a high relapse risk after frontline therapy are generally unsuitable to predict outcome following relapse. A short latency period between diagnosis and recurrence and involvement of multiple locations at relapse identify a population with particularly unsatisfactory results.

*Supported by Deutsche Krebshilfe*

**Post relapse survival in 159 patients with osteosarcoma of the extremity**

S. Ferrari, A. Briccoli, S. Rimondini, M. Mercuri, P. Picci, A. Tienghi[1], A. Brach Del Prever[2], A. Comandone[3] & G. Bacci

*(Dipartimento di Oncologia Muscolo-Scheletrica Istituto Ortopedico Rizzoli; [1]Oncologia Medica Ravenna; [2]Clinica Pediatrica Università di Torino; [3]Oncologia Medica Ospedale, Gradenigo Torino, Italy)*

An analysis of prognostic factors for survival was performed in 159 patients with osteosarcoma of the extremity who relapsed from 1986 to 1997. They had received a combined treatment with surgery of the primary lesion and neoadjuvant chemotherapy delivered according to four protocols based on high-dose methotrexate, doxorubicin, cisplatin and standard dose ifosfamide. The median relapse-free interval (RFI) was 23.6 months (1.9–98.3 months). One hundred and twenty-three (77%) patients relapsed only with lung metastases (monolateral 77, bilateral 46; median number of lung nodules, 3), 36 (23%) patients had recurrence also in other sites; 111 (70%) patients were free of disease after adequate surgical treatment; 85 (53%) patients received a second line chemotherapy treatment, based on high-dose Ifosfamide (15 g/m$^2$, 5-day continuous infusion) in 50 (59%) of them. The estimated 5-year survival after relapse (S) was 27.5%. Univariate analysis identified the site of relapse (lung 5-year S 32%, other 12%, $p < 0.003$), RFI (≤24 months 5-year S 13%, > 24 months 5-year S 46%, $p < 0.001$), the site of lung metastases (monolateral 5-year S 37%, bilateral 17%, $p < 0.001$) and the number of lung metastases (≤ 2 nodules 5-year S 57%, > 2 nodules 5-year S 10%, $p < 0.0001$) as predictive factors for S. Chemotherapy offered a significant advantage only in patients with > 2 pulmonary nodules ( with chemotherapy 5-year S 17 %, without chemotherapy 5-year S 0%, $p = 0.002$), whereas no significant differences were seen in patients with one/two pulmonary nodules (42 vs. 62%, $p = 0.46$). A longer survival time and a higher 5-year S was seen in patients with other than lung metastases who received chemotherapy (with chemotherapy 5-year S 22%, without chemotherapy 5-year S 0%, $p = 0.002$). In a multivariate analysis including only patients with lung metastases the number of nodules (≤ 2 nodules RR 0.3, 95% CI 0.15–0.49, $p <$ 0.0001) and RFI (≤ 24 months RR 2.18, 95% CI 1.3–3.7, $p < 0.005$) were predictive factors for S. About 25% of patients with recurrent osteosarcoma can be rescued. Lung location, a > 24-month relapse-free interval, ≤ 2 pulmonary nodules are positive predictive factors which identify patients who can be treated with surgery only. More than two pulmonary nodules and other than lung location identify poor risk patients who can have benefit from chemotherapy.

**Radiation induced bone sarcoma: a review of the EMSOS database**

H. Patterson & J.S. Whelan on behalf of EMSOS

Data were sought from six centres; 65 patients (31 male and 34 female) were identified who satisfied the criteria for the diagnosis of radiation induced bone sarcoma. The median age at diagnosis of the condition for which the patient received their radiotherapy was 20 years (range 3 months to 66 years), and the most frequent diagnoses were Ewing's sarcoma (13 patients), giant cell tumour (eight patients), breast cancer (seven patients), Hodgkin's lymphoma (five patients), retinoblastoma (four patients) and primary bone lymphoma (three patients). The median age at development of the radiation induce bone sarcoma was 33 years (range 4–78 years) following a median latency of onset of 116 months (range 45–389 months). The radiation dose varied between 12 and 70 Gy, with a median of 47 Gy. The histological diagnosis of the radiation-induced bone sarcoma was osteosarcoma in 40 patients, MFH in 10 patients, fibrosarcoma in nine patients and sarcoma NOS in the remaining six patients. With a median follow-up of 23 months (range 2–208 months), 24 patients are reported to be alive and disease free, 35 patients have died of their radiation-induced tumour, two patients are alive with disease, one patient has died of unrelated causes, and three patients have been lost to follow-up. Of the 24 patients who are alive and well, median follow-up 54 months (range 8–208 months) 12 patients underwent amputation, four patients had a claviculectomy, six patients underwent endoprosthetic replacement, one patient had a craniofacial excision and one patient had a marginal hemi-vertebrectomy. Seventeen patients received combination chemotherapy based on combinations of doxorubicin, cisplatin, methotrexate and ifosfamide, including 10/12 patients who underwent conservative surgery, median follow-up 57.5 months (range 17–208 months).
*Conclusion:* Complete surgical excision in the absence of metastatic disease carries the best prognosis for patients with radiation-induced bone sarcoma. However, salvage appears possible for a proportion of patients who undergo more conservative surgery in conjunction with combination chemotherapy.

**Secondary malignancy following therapy for sarcoma in the COSS, CWS, or CESS studies**

S. Bielack, T. Pilz, B. Fröhlich, B. Kempf-Bielack, S. Ahrens, E. Koscielniak, M. Kevric, M. Paulussen, W. Schwager, J. Treuner & H. Jürgens
*(Depts. of Pediatric Hematology/Oncology, University Muenster and Olgahospital Stuttgart, Germany)*

*Introduction:* The databases of the cooperative soft tissue sarcoma (CWS), Ewing's sarcoma ((EI)CESS), and osteosarcoma (COSS) studies were combined in order to learn more about secondary malignancy after treatment for sarcoma.
*Patients and methods:* All patients with sarcomas entered into any neoadjuvant study of the aforementioned groups prior to 1998 were evaluated for the subsequent development of secondary cancers. Affected patients were analyzed for patient-, tumor-, and therapy-related variables, as well as outcome.
*Results:* Follow-up information was available for 5176 patients, of whom 1996 had been followed >5, 618 >10 , and 103 >15 years. Sixty-two secondary cancers were identified, four in patients with multiple malignancies. The median age at diagnosis of sarcoma had been 11 (0.3–53) years; histology, osteo- 20, rhabdo- 18, Ewing's 18, other sarcoma six. Treatment had included chemotherapy in 61, radiation in 32, and surgery in 51. Secondary malignancies were 33 hematological neoplasms (ANLL/MDS 25, ALL/NHL seven, Hodgkin's disease one) a median of 42 (5–113) months after sarcoma and 29 solid tumors (second sarcoma 12, carcinoma 10, brain tumor five, melanoma one, germ cell tumor one) after 82 (13–177) months. The cumulative risk to develop any secondary malignancy (hematological/solid) was 0.8% (0.6/0.2%) at 5 years, 3.2% (1.8/1.5%) at 10 years and 6.6% (2.0/4.7%) at 15 years. Among patients with secondary cancers, hematological neoplasms were more frequent after Ewing's sarcoma, after metastatic disease, after multiple cytostatic therapies or high-dose chemotherapy, and after epipodophyllotoxins. At least two-thirds of the 24 solid tumors following bone and soft tissue sarcomas other than Ewing's belonged to cancers associated with the Li–Fraumeni syndrome. Actuarial survival at 2 and 5 years from diagnosis of secondary cancer was 48 and 38%. At last follow-up, 29 affected patients survived (20 in complete remission of all malignancies) and 30 had died at a median of 6 (0–79) months after diagnosis of the secondary malignancy (26 due to secondary malignancy, three due to original sarcoma, one due to yet another malignancy), three patients were without follow-up. Prognosis was better for solid tumors than for hematological neoplasms.
*Conclusion:* Secondary malignancies constitute a relevant but not universally fatal threat to patients with sarcoma. Both induction and individual predisposition seem to contribute to their development.
*Supported by Deutsche Krebshilfe & EU-Biomed*

**The role of radiotherapy in ccombination with surgery in 66 Ewing's**

M. San-Julián, J.L. Barroso, J, Vasquez, Azinovic, L. Sierrasesúmaga & J. Ca adell
*(Dpto. Traumatología, Clínica Universitaria, Avda. Pio XII, 36, 31008 Pamplona, Spain)*

*Aim:* To evaluate the role of radiotherapy in combination with surgery in 66 Ewing's sarcomas completely treated at our institution between 1981 and 1997.
*Methods:* The protocol of treatment consisted in preoperative chemo- and radiotherapy (3000–5900 rads), conservative surgery and intraoperative radiotherapy, and postoperative chemotherapy during 1 year. The mean interval between biopsy and surgery was 5 months (1–21). The minimum follow-up was 2 years (2–19).
*Results:* 100% of necrosis or only focii of viable cells were found in 51/66 of cases when comparing with 3/21 patients treated at other institutions with different protocols, who came to our centre after detection of recurrences. Despite the number of 'inadequate' surgical margins (31), only two local recurrences were found in this series (one of them after previous surgery with wrong diagnosis and the other one after insufficient resection and poor necrosis. Complications (nerve palsy, skin necrosis fractures, deformities) due to the use of radiotherapy were found, but only two of these cases required amputation 63% are alive at a mean follow-up of 9 years.
*Conclusion:* Taking into account the number of axial location (17 cases), the difficulties in resection, and the volume of the majority of these tumours, the combination of chemo- and radiotherapy, may improve the possibilities of conservative surgery and the survival rate.

**Pelvic Ewing tumors (ET)—the impact of surgery**

R.W. Rödl[1], Ch. Hoffmann[1], N. Lindner[1], H. Jürgens[2] & W. Winkelmann[1]
*(Departments of [1]Orthopedics and [2]Department of Pediatric Hematology and Onkology, Westfälische Wilhelms Universtät, Münster, Germany)*

*Objective:* Local therapy of pelvic Ewing's tumors (ET) has remained a challenge due to its central localization and the usually large tumor volumes. The impact of surgery for event-free survival is still discussed.
*Method:* 38 patients underwent wide resection of pelvic ET between 1981 and 1996 in a single institution. Chemotherapy was administered according to the appropriate EI/CESS protocols. Radiotherapy was added in 36 of 38 patients. The outcome of these patients were compared to all patients ($n$=113) of the EI/CESS—study who underwent radiotherapy for local tumor control between 1981 and 1996. Both groups were comparable regarding tumor volume. Fifty-nine of 113 radiated patients had metastases at diagnoses compared to 11 of 38 patients, who underwent surgery. Event-free survival curves were estimated according to the Kaplan–Meier method. The log rank test was used to compare the survival curves of both groups.

*Results:* Mean follow-up was 69 month in both groups. Two patients developed local recurrence after surgery (5%) compared to 25 patients (22%) of the radiotherapy group. The event-free survival rate was 39% with surgery and 25% without surgery ($p$ = 0.0532). According to the subgroup of localized pelvic ET, event-free survival rate was 43% with surgery and 28% without surgery ($p$ = 0.1171). Pelvic tumors with metastases at diagnoses show no difference according to local therapy (27 and 23% estimated survival).
*Conclusion:* Wide resection of pelvic ET improves local control significantly. Better local tumor control, performing tumor resection, shows a trend towards better event-free survival. More effective systemic treatment is needed for metastatic patients before they may benefit from surgery. Surgery has to be considered for local therapy in localized pelvic ET.

**Local control in Ewing tumours of bone. A report of the (European Intergroup) Cooperative Ewing's Sarcoma Studies**

M. Paulussen[1], S. Ahrens[1], G. Amann[2], J. Dunst[2], U. Exner[2], B. Fröhlich[1], R. Kotz[2], A. Schuck[2], W. Winkelmann[2], A. Zoubek[2] & H. Jürgens[1]
*(Departments of [1]Paediatric Haematology and [2]Oncology, University of Münster, Münster, Germany)*

*Introduction:* Local recurrence represents a major obstacle towards cure for most sarcoma patients. Local control was analysed for Ewing tumour patients registered in the German/Continental (EI)CESS database.
*Patients and Methods:* On May 1st, 1999, 672 patients with localised Ewing tumours of bone were registered in the three consecutive studies CESS 81 ($n$=95), CESS 86 ($n$=213), and EICESS 92 ($n$=364). Four hundred and six patients (60%) were male; median age was 14 (range, <1–35) years. Primary tumour sites were: pelvic ($n$=145, 22%), other trunk ($n$=179, 27%), femur ($n$=127, 19%), or other extremity ($n$=215, 32%) (incomplete data in three patients). All patients received neoadjuvant systemic multiagent chemotherapy and local therapy by surgery ($n$=145, 23%), radiotherapy ($n$=151, 24%), or combined surgery and radiotherapy ($n$=332, 53%) (incomplete data in 44 patients). Local control was assessed by means of competing risk analyses, event-free survival (EFS) was computed according to Kaplan and Meier. Comparisons between groups were done by $\chi^2$- or logrank-tests.
*Results:* After a median time under study of 93 (range, 1–222) months, 5-year EFS was 0.61. The probability of local (including combined local plus distant) failure (LOCFAIL) was 0.11. Local control has improved more recently: CESS 81, LOCFAIL 0.25, CESS 86, 0.09, EICESS 92, 0.08; $p$<0.01. Pelvic (LOCFAIL 0.17) or other central axis sites (0.15) bear a higher risk of local failure than femur (0.04) or other extremity (0.08) sites, $p$<0.01. Local failures were less common when surgery was part of the local therapy strategy: LOCFAIL surgery, 0.04, radiotherapy, 0.28, combined surgery plus radiotherapy, 0.07; $p$<0.01.
*Conclusion:* Local control in Ewing tumours is sufficient with modern local therapy. Complete surgical resection should be attempted where feasible, and eventually be supplemented by radiotherapy.
*Supported by Deutsche Krebshilfe, EU Biomed, and Sabrina Forschungsfond e.V.*

**The role of radiotherapy in multimodality treatment of Ewing's sarcoma**

A. Cassoni, J. Whelan & A. McTiernan
*(University College London Hospitals, London, UK)*

*Material and methods:* Between 1990 and 1999, 83 patients with Ewing's/PNET tumours were treated by radiotherapy with radical intent as part of multimodality therapy as the only local therapy (48), or as an adjuvant to surgery (35). Their median age was 18 years (range 5–54); minimum follow-up is 6 months. Primary sites include pelvis 25; lower limb 30; chest wall five; spine five. Twenty-one had metastases at presentation. Doses of radiation were 55 Gy as sole therapy, 45 Gy as adjuvant (but doses to spinal cord, heart and large volumes of lung were limited). In 69 a hyperfractionated regimen, concurrent with chemotherapy, was used (twice daily 1.6-Gy fractions with planned gaps); in the remainder conventional once daily treatment in 1.8–2-Gy fractions
*Results:* There was failure of local control in six of those treated with adjuvant radiotherapy; all were concurrent with or, preceded by, distant progression and all these patients are dead. A further seven have died without evidence of local relapse. Of the 48 with radiotherapy as the sole local therapy, 14 had failure of local control. In nine this was within 6 months and associated with progression elsewhere. All these patients are dead. Five patients experienced late and, initially, isolated local relapses 10, 25, 58, 60 and 24 months after radiotherapy. Three of these have died 24, 22, and 16 months after local relapse. A further 14 have died without evidence of local relapse. The overall control rate is 83% in the adjuvant group (overall survival of group 63%) and 71% in the sole local therapy group (OS 56%).

**Local recurrence after treatment of Ewing's sarcoma**

A. Abudu & R.J. Grimer
*(The Royal Orthopaedic Hospital Oncology Service, Bristol Road South, Birmingham B31 2AP, UK)*

*Purpose:* To ascertain the prognosis and significance of local recurrence following treatment of Ewing' sarcoma.
*Material:* 300 patients with non-metastatic Ewing's sarcoma have been treated since 1975. Of these, 25 have developed local recurrence at some time in the course of their disease.
*Results:* Of the 30 patients, 15 had undergone surgical treatment and 10 radiotherapy alone. All had received chemotherapy. The most common site for local recurrence was in the pelvis, followed by the proximal femur. The time to LR averaged 19 months, but was only 14 months in those treated by radiotherapy alone whilst was 26 months in those treated surgically. In four cases the LR was the first evidence of recurrent disease, whilst in a further 13 cases further disease was discovered either at the time of restaging or within a few months. The LR was treated by further chemotherapy and radiotherapy in most cases but two underwent amputation. Original treatment, response to chemotherapy and type of surgery made no significant difference on the outcome of LR. In patients with previous or synchronous metastases, all were dead in a further 14 months (median survival 5 months), whilst in those without metastases at the time of restaging there was a 28% chance of survival at 4 years.
*Conclusion:* LR in Ewing's is bad news. Most will have metastases develop within a short time and will succumb to this recurrent disease. In this series there were no patients with late isolated LR following previous radiotherapy. Control of the LR can usually be achieved with further radiotherapy, treatment of the metastases requires systemic treatment but finding this form of effective treatment is proving difficult.

**Preliminary results of the EICESS 92 study**

H. Jurgens[1], S. Ahrens[1], B. Frohlich[1], M. Paulussen[1], A. Zoubek[2], R. Kotz[2], G. Amann[2], I. Lewis[2], P.A. Voûte[2], W. Winkelmann[2], B. Dockhorn-Dworniczak[2] & A.W. Craft[2]
*(Departments of [1]Paediatric Haematology and [2]Oncology, University Children's Hospital, Munster, Germany)*

The disease-free survival for patients with primary Ewing tumours of bone has impressively improved with the introduction of systemic chemotherapy in addition to local control with surgery and/or radiation. EICESS 92, a project of the German (GPOH) and British (UKCCSG) societies for paediatric oncology, has stratified treatment in standard risk (SR) and high risk (HR) based on tumour volume (</_100 ml). SR patients were randomised for cyclophosphamide (CYC)- versus ifosfamide (IFO)-based maintenance following induction with vincristine, actinomycin D, ifosfamide, and adriamycin (VAIA). HR patients were randomised for VAIA versus the addition of etoposide (EVAIA). The trial was open from July 1992 until November 1999. A total of 470 protocol patients with localised disease and no visible metastases at diagnosis were recruited: SR, 147 patients; HR, 323 patients. The median age was 15 years, ranging from 8 months to 35 years, the female/male ratio was 1:1.5. The median time since diagnosis was 46 months, ranging from 3 to 88 months. On 15 November 1999 the estimated EFS at 5 years was 63% for the total group, and 75% for SR and 58% for HR patients. With respect to randomisation, the 5-year EFS for SR patients was 71% for VACA and 79% for VAIA ($p$=0.37). In HR patients the results were 54% for VAIA and 62% for EVAIA ($p$=0.60). Regarding local therapy, 75% of patients underwent surgery, 24% surgery alone and 51% in combination with either pre- or postoperative radiation; 23% of patients received definitive radiation. EFS at 5 years according to local therapy was 67% for surgery with or without radiation, and 54% for definitive radiation. The local failure rate was 3.6% for the total group, 2.3% for patients with surgery +/– radiotherapy, and 8.4% for patients with definitive radiation. Regarding response to treatment in HR patients, the proportion of good histological response (<10% viable tumour) was 72% following VAIA and 80% following EVAIA ($p$=0.20). No secondary malignant neoplasms have been reported so far in this cohort of patients. To date, the results according to randomisation have not yielded statistical significance. Longer follow-up is needed to draw definitive conclusions. Surgery is superior in comparison to radiation to assure safe local control.

*With support by Deutsche Krebshilfe (M43/92/Jü2), EC-BIOMED 1 (BMH1-CT92-1341), EC-BIOMED 2 (BMH4-98-3956)*

**Limb-salvage surgery in Ewing's sarcoma of childhood: 20 years experience at Istituto Ortopedico Rizzoli**

M. Manfrini, M. Mercuri, P. Rosito[1], E. Barbieri[2], S. Giacomini, L. Campanacci & G.Bacci

*(Istituto Ortopedico Rizzoli, [1]Clinica Pediatrica and [2]Istituto di Radioterapia, Bologna, Italy)*

In the period 1978–1997, 71 children affected by non-metastatic Ewing sarcoma of bone were surgically treated at Istituto Ortopedico Rizzoli. There were 35 female and 36 male patients, all under 13 years of age (mean age 8.2 years). All patients received chemotherapy according to the different historic protocols. A limb-salvage approach was preferred in 58 cases (82%), 37 of whom had a skeletal reconstruction. Rotation-plasty was performed in four patients (5%), while the remaining nine cases were amputated (13%). Local radiotherapy was delivered in eight children (in three patients before and in five patients after surgery). Surgical indications changed along the years. Classic amputations represented 21% (5/24) of surgical treatments during the first decade 1978–87, while in the latter one (1988–97) they decreased to 8% (4/47). An opposite trend was seen both in rotation-plasties (from 0 to 8%) and limb-salvage surgeries (from 79 to 84%). In the 37 cases where the bone was reconstructed, the tumor was localized mainly in diaphyseal femur (10 cases), proximal femur (six) and proximal humerus (seven) followed by diaphyseal tibia (four), pelvic bones (four), proximal tibia (two), distal femur (one), diaphyseal (one) and distal radius (one), first metatarsus (one). Reconstructive techniques as well have improved in the last 20 years: in the period 1978–87 the most common reconstruction was represented by artificial implants (77%), while in the next decade, the main surgical group was that of biological reconstructions (85%). Three patients presented a local recurrence at a mean time of 43 months after surgery (range 10–62 months). In a girl treated by resection of the fibula plus radiation therapy, a treatment related sarcoma appeared after 72 months. At a mean time of 85 months (range 25–241 months) from surgery, two cases were lost to follow-up, 50 patients were continuously disease free (70%), 14 died of disease and five were alive after treatment of a relapse (one local, four distant). In reconstructed patients, 43% needed secondary surgery because of septic or mechanical complications; four children (11%) underwent amputation for deep local infection. In the reconstructed group, 32 patients with more than a 5-year follow-up were available for functional evaluation according MSTS: 72% presented a satisfactory result (excellent or good), 16% fair, and 12% poor.

**Long-term follow up of Ewing's sarcoma patients**

F. Mittermayer, M. Sluga, R. Windhager[1], P. Krepler, S. Lang, H. Heinzl, M. Dominkus & R. Kotz

*(Department of Orthopaedics, University of Vienna, Währinger Gürtel 18–20, A-1090 Vienna, Austria and [1]Department of Orthopaedics, University of Graz, Austria)*

This study analyses significant prognostic factors, interdisciplinary approach and development of therapy of Ewing's sarcoma patients over the last three decades. Between 1/1949 and 12/1994, 140 patients with Ewing's sarcoma were treated at our institution. Median follow-up was 8.5 years. Age at the time of diagnosis ranged between 1 and 51 years (mean 16 years). Fifty-one tumors were located within the trunk and 89 within the extremities; 21 patients had preoperative metastases; 55 patients were treated in the period between 1949 and 1980 (group 1), 85 patients between 1980 and 1994 (group 2). Patients in group 1 were either treated without ($n$=5) or with polychemotherapy ($n$=47). Patients in group 2 were treated by CESS ($n$=50) or EICESS ($n$=35) chemotherapy. In group 1, 23 patients underwent only biopsy, 32 patients had definitive surgical treatment, 48 patients received either only or additional radiation therapy. In group 2, the respective numbers of patients were: seven, 78, 43. Five-year overall survival increased from 27 to 57%, 5-year MFS from 25 to 59%. Univariate independent significant prognostic factors on overall survival were preoperative metastases ($p$=0.0001), tumor localisation ($p$=0.0048), type of chemotherapy ($p$=0.002) and response to chemotherapy ($p$=0.0004). In multivariat analyses the developement of chemotherapy ($p$=0.037) was evaluated as significant factor for survival, furthermore tumor localisation ($p$=0.0017) and metastases at diagnosis ($p$=0.0025). Systemic therapy rather than type of local treatment seems to influence the overall survival. There was no significance found either in univariate or in multivariate analyses in radiation therapy and/or surgical treatment.

**A single centre experience of an intensive induction therapy (VIDE) including stem cell collection for Ewing's family of tumours (EFT)**

S.J. Strauss, A. McTiernan, D. Driver, M. Hall-Craggs, A. Sandison, A.M. Cassoni, A.M. Kilby, M. Michelagnoli, J. Pringle, J. Cobb, J. Witt, S. Cannon, T. Briggs & J.S. Whelan

*(The London Bone and Soft Tissue Tumour Service, The Middlesex Hospital, Mortimer Street, London W1N 8AA, UK)*

*Aim:* To examine the feasibility, tolerability and toxicity of an intensified induction regimen (VIDE) in patients with newly diagnosed EFT; to assess ability to maintain dose intensity, and predictability of peripheral blood stem cell mobilisation.
*Patients and methods:* Thirty patients, 10 with metastatic disease at diagnosis, were treated with vincristine 1.4 mg/m$^2$ (max. 2 mg) on day 1, doxorubicin 20 mg/m$^2$, ifosfamide 3 g/m$^2$ + mesna, and etoposide 150 mg/m$^2$ on days 1–3. Cycles were given every 21 days for up to six cycles. Patients with localised disease received further chemotherapy with vincristine 1.4 mg/m$^2$ on day 1, actinomycin 0.75 mg/m$^2$ and ifosfamide 3 g/m$^2$ on days 1 and 2 (up to eight cycles). Those with metastatic disease at diagnosis were considered for high dose therapy with busulphan (600 mg/m$^2$) and melphalan (140 mg/m$^2$) with stem cell rescue. Treatment response was evaluated clinically and radiologically. On completion of induction therapy, surgery or radiotherapy was undertaken.
*Results:* The median age was 17 years (7–36). One hundred and seventy cycles of VIDE were given. The median treatment interval was 21 days (21–60) and nadir count, Hb 8.3 (6.3–11.9), neutrophils 0.045 (0.0–2.1) and platelets 45 (3–343). Grade 3–4 neutropenia was seen in 97% of cycles and grade 3–4 thrombocytopaenia in 54%. There were 103 episodes of febrile neutropenia (61%). Growth factor support reduced infectious complications by 33%. Treatment interval was maintained in 80% of cycles. Ifosfamide dose was maintained in 97% and doxorubicin dose maintained in 98% of cycles. Etoposide dose was reduced, or omitted, in 24% of cycles. Four patients did not complete six cycles due to unacceptable toxicity and one patient progressed on treatment. Twenty patients underwent peripheral blood stem cell harvesting, all successfully, 15 after cycle 3, and five after cycle 4. Median CD34 yield was 3.6 × 10$^6$/kg (0.1–14.5). Overall response to treatment, measured in 21 patients, was 86%. Histological response was measured in 11 patients undergoing surgery, with seven patients (64%) achieving > 90% necrosis of tumour.
*Conclusion:* VIDE is an effective induction regimen with substantial but acceptable toxicity that allows predictable mobilisation of stem cells in all patients. Maintenance of dose intensity is feasible in the majority of patients for six cycles, and treatment interval sustainable. Growth factors play a role in maintaining dose intensity and reduce the rate of infectious complications.

**A phase II study of docetaxel for the treatment of recurrent osteosarcoma and Ewing's tumours**

J.S. Whelan & A. McTiernan
*(London Bone and Soft Tissue Tumour Service, The Middlesex Hospital, London, UK)*

*Study aims:* To determine the response to and toxicity of Docetaxel in (a) recurrent osteosarcoma and related spindle cell tumours of bone (OS) and (b) recurrent round cell tumours (Ewing's tumours and primitive neuroectodermal tumours—EFT).
*Background:* Better systemic therapies are required to improve survival from OS and EFT. However few new active agents have been identified. The taxanes, taxol and docetaxel, have shown important activity, alone and in combination, in other diseases such as breast, ovarian and lung cancer but their value in OS and EFT is unknown.
*Subjects and methods:* Eligibility criteria included a histological diagnosis of EFT or OS, measurable progressive or recurrent disease after chemotherapy and normal liver function. Docetaxel 100 mg/m$^2$ was given as a 1-h infusion every 3 weeks. Response was assessed after every two cycles to a maximum of six. Nine patients with osteosarcoma and one with MFH were treated. All except one had extremity tumours treated by surgery. Eight had received initial chemotherapy with doxorubicin and cisplatin, one with the addition of ifosfamide and one a multiagent regimen. Five had been treated with second line chemotherapy before receiving Docetaxel. Nine had lung metastases and one intra-abdominal disease. Fourteen patients with EFT were treated. Three had extraosseous primaries. Six had other chemotherapy at relapse before docetaxel. Five had received high dose therapy.
*Results:* Patients received a median of two cycles (range 1–6). There were no responses in patients with OS. A single partial remission was seen in EFT (response rate 7%). One other patient had stable disease. A total of 61 cycles were given. Toxicity included alopecia and myelosuppression. Grade 3/4 infection was uncommon when growth factors were used.
*Conclusion:* Docetaxel does not appear to have major activity as a single agent in this heavily pre-treated group of patients with recurrent OS and EFT. Window studies should be considered in EFT and the combination of docetaxel and cisplatin or carboplatin could be investigated in OS.

**Treatment of soft tissue sarcomas across a region**

J. Glencross[2], P. Silcocks[3] & M.H. Robinson[1]
*([1]YCR Department of Clinical Oncology, Weston Park Hospital, [2]Leicestershire Health Authority, [3]Trent Cancer Registry, Weston Park Hospital)*

*Background:* Bone and soft tissue sarcomas are a rare and heterogeneous group of tumours, rarely seen by most clinicians during their careers. Early recognition, appropriate and timely investigations, and treatment are shown to markedly affect outcomes. In Trent Region a soft tissue sarcoma interest group had developed outline consensus based guidelines for investigation and management. The Trent Cancer Registry was asked to undertake a retrospective baseline audit of the care of such patients in Trent.
*Findings:* Soft tissue sarcomas registered with Trent Cancer Registry in 1995–1997 and meeting ICD-O criteria for site and morphology were selected. Data were collected from clinical records against a set of criteria outlined in the protocol in all registering hospitals. Data included: recording of clinical details, specialties involved in management, diagnostic and staging investigations, completeness of surgery, histological reporting, use of adjuvant therapy and outcome (death or known recurrence). From 257 registrations, 204 cases were included: 10% of notes were unobtainable; 50% were initially referred to general surgeons, although definitive surgery was performed by surgeons (40%) and orthopaedic surgeons (33%); 74% saw an oncologist; 55% had a diagnostic CT or MRI scan. Only 52% had adequate staging investigations; 63% had a biopsy; but 13% were 'shelled out' at biopsy. Thirty-five percent had wide or adequate excision; in 48% primary surgery was incomplete or marginal. Outcome was poorer in these patients. Histological reporting was variable. No consistent grading system was used; details on tumour margins were available for 66% of cases; tumour size stated in 73% of cases; 52% received adjuvant therapy; 10% being considered for EORTC trial entry. Follow up was generally be a specialist oncology team, although 10% had no recorded follow up.
*Conclusions:* The audit demonstrates that optimal care as defined by the soft tissue sarcoma group in Trent was not provided over this period within the region. All clinicians should follow management guidelines and proposals to set up specialist referral centres should be considered.

**Limb salvage surgery for soft tissue sarcomas of the hands and feet**

R. Capanna, P. Caldora, D.A. Campanacci, G. Beltrami, M. Ceruso, M. Innocenti, L. Frazzetta & P. Olmi[1]
*(C.T.O. and [1]Radioterapia, Firenze, Italy)*

Soft tissue sarcomas arising in the hand–wrist and foot–ankle regions are frequently extracompartmental. In these locations, adequate surgical margins can only be obtained by the sacrifice of several anatomical structures, including skin, tendons, vessels, and nerves, therefore amputation has always to be considered as a treatment. However, by the use of microsurgical and plastic surgical techniques and of advanced reconstructive procedures, limb salvage with wide surgical margins is feasible in many patients. Moreover, sarcomas in these sites have often a superficial location and their diameter is frequently <5 cm, both being considered as favorable prognostic features. This series includes 51 patients with a soft tissue sarcoma of the hand (12), wrist (nine), foot (21), and ankle (nine) operated in the last decade. Tumors were high-grade lesions in 43 cases, and low-grade lesions in eight cases, and all but two cases had an extracompartmental presentation. The diameter of the primary tumor was <5 cm in 43 cases, and >5 cm in eight cases. The lesion was primary in five cases, recurrent in 14 cases, while a radicalization of a previous inadequate surgery was done in 32 cases. Five patients (10%) were amputated, whereas limb salvage surgery was feasible in the remaining 47 cases. Of these, 43 cases had wide margins, three marginal margins, and one intralesional margins. Either rotational (nine) or free (33) flaps were required for reconstruction in 42 cases. Muscular transposition or tendon grafts were used in 12 cases, nerve grafts in two cases, joint arthrodesis in four cases, osteoarticular allografts in one case, and an index–pro-thumb transposition in one case. At an average follow-up of 60 months (4–153), 37 patients were continuously disease-free (72.5%), five had local recurrences (10%), and 13 developed metastases (25%). In our experience, soft tissue sarcomas of the hands and feet could be treated by limb salvage surgery in 90% of the cases, whereas secondary amputation due to local recurrence was needed in three cases (6%).

**Bone marrow analysis by RT-PCR of newly diagnosed Ewing tumour patients: a prognostic factor?**

B. Fröhlich[1], S. Ahrens[1], M. Paulussen[1], H. Jürgens[1] & B. Dockhorn-Dworniczak[2]
*(Departments of [1]Paediatric Oncology and [2]Pathology, University of Münster, Münster, Germany)*

*Study and aim:* More than 98% of Ewing tumours (ET) are characterised by a translocation of chromosome 22, leading to an EWS/ets-oncogene fusion. In recent studies bone marrow (BM) positivity detected by reverse transcriptase-polymerase chain reaction (RT-PCR) has been considered as an adverse prognostic factor for event free survival (EFS). Furthermore the chimeric transcript 7/6 has been proposed to be associated with a better clinical outcome than other transcription factors.
*Patients and methods:* Between 04/1993 and 04/1998, newly diagnosed ET of 100 patients (patients) were analysed for Ewing-specific rearrangements. BM was available for RT-PCR in 62 patients: 34/62 patients with localised ET, 28/62 patients with primary metastatic ET. Results are demonstrated by Kaplan–Meier analysis.
*Results:* BM positivity was detected in 11/31 (32%) patients with localised disease and in 23/28 (82%) patients with primary metastastic ET. In localised ET, EFS 4 years after diagnosis is 0.80 in patients without RT-PCR BM contamination and 0.71 in those with BM involvement ($p$=0.5241). Five primary metastatic patients who had no RT-PCR BM contamination showed an EFS 3 years after diagnosis of 0.67, compared to 0.19 in patients with BM positivity ($p$=0.0088). Subgroup analysis according to type of chimeric transcript shows, that there is no significant difference in outcome between ET fusion transcript 7/6 compared to other ET transcripatients: 4-year EFS is 0.50 in both, ET with chimeric transcript 7/6 and ET with other transcripts ($p$=0.6771).
*Conclusions:* At present there is no evidence for an independent prognostic impact of RT-PCR BM positivity in localised ET. The type of chimeric transcript does not seem to correlate with outcome. Prospective studies in larger groups of patients are needed to fully evaluate the prognostic significance of molecular findings in ET.

*Supported by Deutsche Krebshilfe Grant M 43/92/Jü 2, EC BIOMED 1 and 2 Grants BMH1-CT92-1341 and BMH 4-98-3956, and the Sabrina Forschungsfond e. V.*

**Soluble Fas-antigen (SFAS) in the serum of bone tumour patients**

N. Kushlinsky[1], S. Abbasova[2], Yu. Solovyev[1], I. Kostanyan[2], I. Babkina[1], A. Izgorodin[3], V. Lipkin[2] & N. Trapeznikov[1]
*([1]Russian N.N.Blokhin Cancer Research Centre RAMS; [2]M.M.Shemyakin & Yu.V.Ovtchinnikov Institute of Bioorganic Chemistry RAS; [3]N.N.Burdenko Main Military Clinical Hospital of the Ministry of Defence of RF, Russia)*

Apoptosis is one of the important physiological processes which disturbances underlie many pathological states including tumour formation. We have developed a sandwich-type enzyme-immunoassay test system for quantitative determination of one of the key receptors triggering cellular apoptosis (Fas-Fas/APO-1/CD95) in the blood serum of practically healthy people and bone tumour patients (sFas). A comparative analysis of sFas incidence and levels in these two groups was performed. Eighteen positive hybrids that reacted with Fas were received, and nine most active clones were selected for further experiments. Antibodies were produced in the ascitic fluid of 8-week-old BALB/c mice. Full-size recombinant baculoviral Fas ('Coultronics', France) was used as the antigen. The characteristics of the obtained antibodies included the following steps: determination of the classes and subclasses of immunoglobulins, the types of their light chains, measurement of the association constants of antigen–antibody complexes, and evaluation of the possibility of applying the antibodies received for Western blot analysis. The lower limit of sensitivity of the developed system comprised 0.52 ng/ml. Serum samples of 108 bone tumour patients (aged 16–67 years) were studied: osteosarcoma (45), chondrosarcoma (25), Ewing tumour (12), giant-cell tumour (12), malignant fibrous histiocytoma (14); benign and tumour-like bone lesions (osteochondroma, one; osteoblastoma four; chondroblastoma two; anevrismal bone cyst, three); mechanical bone traumas (18). Control group consisted of 116 practically healthy people of comparative age (65 men and 40 women). sFas was more frequently found in the serum of oateosarcoma (85.7%) and Ewing tumour (100%) patients than in the serum of healthy people (36%). The mean levels of sFas were higher in osteosarcoma (3.72±0.89 ng/ml), Ewing tumour (8.32±3.35 ng/ml) and chondrosarcoma (4.31±1.09 ng/ml) patients than in the control group (0.86±0.30 ng/ml) and in patients with mechanical traumas (2.62±0.50 ng/ml). Possible clinical value of sFas and its association with disease pathogenesis and common biochemical and clinico-morphological criteria of prognosis in bone tumour patients is discussed.

**Assessment of the role of macrophage osteoclast differentiation in the pathogenesis of tumour osteolysis**

*T.T. Yang, A. Sabokbar, C.M.L. Gibbons & N.A. Athanasou*

*(Nuffield Orthopaedic Centre, University of Oxford, Oxford OX3 7LD, UK)*

Osteolysis is a major complication of both benign and malignant tumours that arise in bone. The cellular mechanisms that account for such bone destruction are poorly understood. Osteoclasts are specialized bone-resorbing cells which effect the bone resorption associated with tumour osteolysis. It has recently been shown that tissue macrophages are capable of differentiating into bone-resorbing osteoclastic cells and that macrophage colony-stimulating factor (M-CSF) and osteoprotegerin ligand (OPGL), an osteoblast-associated factor, are required for this to occur. The main aims of this study were to determine: (i) whether macrophages isolated from benign and malignant mesenchymal tumours are capable of osteoclast formation and bone resorption; and (ii) the cellular and humoral requirements for this to occur. Macrophages were isolated from two soft tissue tumours, a malignant fibrous histiocytoma of the thigh and a benign schwannoma of the saphenous nerve. (Extraskeletal mesenchymal tumours rather than bone tumours were studied to avoid possible contamination by osteoclasts). Macrophage cultures were incubated on glass coverslips and dentine slices which had been previously seeded with UMR106 osteoblast-like cells. A total of $1\dot{1}0^5$ macrophages were added to each well and the co-cultures maintained in the presence of $1,25(OH)_2D_3$, dexamethasone and M-CSF for up to 21 days. Macrophages were also incubated in the absence of UMR106 cells but in the presence of OPGL and M-CSF. Osteoclast formation in these co-cultures was assessed for expression of osteoclast markers, i.e., TRAP and lacunar bone resorption. Macrophages isolated from both tumours, when co-cultured with UMR106 cells in the presence of $1,25(OH)_2D_3$, dexamethasone and M-CSF, were capable of differentiation into osteoclasts as determined by the formation of TRAP-positive multinucleated cells and lacunar resoprtion pit formation. Osteoclasts also formed in the presence of OPGL and M-CSF alone. No evidence of TRAP expression or lacunar resorption was seen when UMR106 cells, OPGL or M-CSF were omitted from macrophage cultures. The results of this study show that macrophages present in benign and malignant mesenchymal tumours are capable of osteoclast differentiation and that both M-CSF and OPGL (expressed on the surface of osteoblasts) are required for this to occur. As reactive macrophages form a major cellular component of most benign and malignant tumours of bone, macrophage–osteoclast differentiation is likely to play a significant role in tumour osteolysis.

**Giant cell tumor of bone: new approaches to further characterize the neoplastic cell origin**

M. Wülling, M. Werner, G. Delling & E. Kaiser
*(Abteilung Osteopathologie/Zentrum für Biomechanik, Universität Hamburg, Hamburg, Germany)*

*Aims:* The giant cell tumor of bone (GCT) is a locally destructive tumor with a high rate of recurrence. Its frequency is 5% of all bone. Histologically the GCT consist of at least three different cell types. The neoplastic, mononuclear cell that is hypothesized to attract a second mononuclear cell, the $CD14^+$ monocyte. The third cell component is the characteristic osteoclast-like, multinuclear giant cell, which forms from the attracted monocytes through tumor-induced cell fusion. The origin of the neoplastic mononuclear cell has not been elucidated. In an attempt to further characterize the nature of the neoplastic component of GCT we utilize mesenchymal and osteoblastic differentiation markers.
*Methods:* Histological diagnosis of five GCT was performed by two specialised pathologist (M. Werner, G. Delling). All three cell types were harvested from representative tumor areas and cultured for further studies. Cells from passages 0–3 were fixed on coverslips for immunocytochemical evaluation using the APAAP method.
*Results:* The expression of vimentin, SH2, SH3, SH4 and CD166 antigens was observed in all neoplastic cells of the five GCT. The surface markers SH2, SH3, SH4, CD166, Stro1, stem cell factor and alkaline phosphatase are expressed on bone marrow stromal cells during osteoblastic differentiation. While the SH2, SH3, SH4 and CD166 antigens are characteristic for the rare mesenchymal stem cell, the markers Stro1 and alkaline phosphatase occur in more differentiated osteoblast precursors. However, none of the stromal cells were positive for the Stro1-antigen, alkaline phosphatase, stem cell factor nor its receptor c-kit. Also, these neoplastic cells showed no histiocytic markers like CD68 and CD14.
*Conclusion:* Based on these data, we suggest that the neoplastic component of GCT originates from the undifferentiated mesenchymal stem cell population, which occurs in a frequency of 0.1% in normal adult bone marrow.
*This work was supported by the Deutsche Forschungsgemeinschaft, GK476.*

**A new P53 germline mutation in a Li-Fraumeni family that induces chemoresistence to doxorubicin**

L. Sangiorgi[1], M.A. Cerone[2], S. Soddu[2], G. Gobbi[1], E. Lucarelli[1], A. Brach del Prever[3], P. Picci[1] & L.J. Helman[4]
*([1]Rizzoli Orthopedic Institute, Bologna, [2]Regina Elena Cancer Institute, Rome, [3]University of Turin, Turin, Italy, and [4]NCI, NIH, Bethesda, MD, USA)*

A phenotypic Li-Fraumeni family (mother died of breast carcinoma at the age of 35, brother died of rabdomyosarcoma at the age of 3, two sisters died of osteosarcoma at the ages of 10 and 14) was investigated for the presence of germline p53 mutations. SSCP for exons 4–11 of the gene on tumor DNA from the two sisters revealed an abnormal conformer in exon 6. DNA sequence analysis showed a transversion from adenine to cytosine at codon 220 (amino acid change from tyrosine to serine). The same pattern was observed on microdissected paraffin-embedded tissue of both the mother and the brother. We generated, by site-directed mutagenesis, a plasmid encoding the $p53^{SER220}$ mutation (pLp53-S220). We transfected fibroblasts from p53 –/– mice (F10) with pLp-S220 and pLp53-H175 (a plasmid encoding the $p53^{His175}$ mutant). The presence of the two mutations did not increase the proliferation rate of F10 fibroblast. Drug sensitivity (assessed by $IC_{50}$ value) of the fibroblasts carrying the mutations was evaluated for doxorubicin, cisplatin and 5-florouracil. Fibroblasts carrying the $p53^{SER220}$ and $p53^{His175}$ mutations showed a selective resistance for doxorubicin with, respectively, a 3.5- and a 2.9-fold increase. These data were confirmed by the evaluation of the clonogenic ability of the fibroblasts carrying the different transfectants. In conclusion, the $p53^{SER220}$ germ-line mutation seems to induce a gain of function in term of chemoresistency for the mutated p53 protein.

**Prognostic role of metalloproteinases in soft tissue sarcomas**

*M.S. Benassi, G. Gamberi, G. Magangoli, M. Merli, L. Molendini, P. Ragazzini, F. Chiesa & P. Picci*

(*Laboratorio di Ricerca Oncologica, Istituti Ortopedici Rizzoli, Bologna, Italy*)
*Aim:* The earliest stage of the process of tumor invasion is an increased permeability to malignant cell movement resulting from an extracellular matrix (E_CM) degradation by proteases. High levels of metalloproteases (MMPs) have been related to cell metastatic potential and, inversely, the presence of specific MMP inhibitors (TIMPs) could suppress metastasis preserving ECM integrity. The aim of this study was to evaluate the prognostic role of MMP2, MMP9 and TIMP2 expression in relation to disease-free survival (DFS) in 73 patients (32 relapsed and 41 disease free) with soft tissue sarcomas.
*Materials:* The tumors included 29 liposarcomas (11 low grade and 18 high grade), 15 malignant peripheral nerve sheath tumors (MPNST), of which four low grade and 11 high grade, and 29 synovial sarcomas (14 spindle cell monophasic and 15 biphasic). The expression and distribution pattern of enzymes was evaluated by immunohistochemistry on paraffin-embedded tissue, while their level was calculated by immunoblotting analysis on fresh tissue.
*Results:* MMP2 reactivity showed a significant correlation with the metastatic event ($p$<0.001) and grading ($p$<0.001), while the lack of TIMP2 expression was a variable for poor prognosis ($p$<0.01). The difference between the DFS curves based on MMP2 and TIMP2 expression was statistically significant ($p$<0.01 and $p$<0.05, respectively).
*Conclusions:* An unbalance in the protease/protease inhibitor system as seen from our data may increase soft tissue tumor aggressiveness. Finding new easily evaluable prognostic factors could improve treatment and subsequently prognosis for these patients.

**Outcome after limb salvage and reconstruction with massive allograft after resection of sarcoma. Results more than 5 years after surgery**

N.J. Lindner, R.W. Rödl, Ch. Hoffmann, G. Gosheger, F. Böttner, T. Ozaki, C. Gebert & W. Winkelmann
*(Orthopaedic Department, University of Muenster, Albert-Schweitzer-Str. 33, 48149 Münster, Germany)*

*Purpose:* Results of reconstructive surgery after five or more years using massive allograft transplantation.
*Material and methods:* One-hundred and fifteen reconstructions with massive allografts were performed in patients with various tumors who underwent limb salvage at a single institution more than five years ago. Among them 33 patients had Ewing's sarcoma, 45 had osteosarcoma, 15 had chondrosarcoma, and two each had malignant fibrous histiocytoma of bone, leiomyosarcoma of bone, synovial sarcoma and 13 had other benign and malignant lesions. All grafts were procured according to the guidelines of the European association of tissue banks and received gamma sterilisation with cobalt-60 25 kGy.
*Results:* Twelve patients had large defects in the humerus, 42 in the femur, 33 in the tibia, 19 in the pelvic region, and nine in other regions. Osteosynthesis consisted of intramedullary rods, AO plate or screw and K wire fixation. Intercalary, arthrodesis and joint reconstrucion was performed. All grafts were longer than 10 cm. Twenty-three grafts were removed due to infection and fracture and revision with another limb salvage procedure was performed. Two patients with pelvic allografts had to be amputated due to infection and local recurrence. Skin problems, type of resection, intrapoperative blood loss, cementation of allografts did not significantly influence the infection rate. The difference between the average tumor volume with and without an early infection was significant. The average volume of allograft with an infection was significantly larger than that without infection. The difference between duration of surgery in patients with and without an early infection was significant.
*Conclusion:* Reconstruction of large bone defects after tumor resection has a high complication rate. Osteosynthesis has to provide a sturdy fixation to protect the graft and allow healing of the junctions to host bone. Once fusion occurs the complication rates are minimal.

**The combination vascularised fibula/massive bone allograft. Imaging analysis at an over 5-year follow-up**

M. Manfrini, D. Vanel, M. Innocenti[1], C. Malaguti, M. De Paolis, M. Ceruso[1] & M. Mercuri
*(Istituto Ortopedico Rizzoli, Bologna and [1]CTO, Firenze, Italy)*

In a homogeneous group of 32 lower limb skeletal reconstructions performed from February 1989 to May 1995, a vascularised fibula autograft (VFA) was inserted into a massive bone allograft (MBA), cut as a trough. Implant biological behaviour was investigated on the basis of serial radiological examinations obtained in 22 patients regularly followed for an average time of 91 months (range 60–132). Mean age was 13 years (range 4–30) and 17 patients received neoadjuvant chemotherapy.
*Methods:* All patients were studied by standard X-ray every 6 months in the first 5 years and then every year; in all cases an average of three CT postoperative evaluations (two to six) at—over 1-year—intervals were performed with the same CT Unit. Computer assisted analysis was performed at three constant levels within the reconstruction, so that the subsequent CT exams could be compared. The following measurements were recorded :VFA Maximal Sagittal and Transverse Diameter, Total and Medullary Area, Maximum Cortical Thickness and Cortical Density; MBA Cortical Thickness and Cortical Density. Parametrical statistical comparison of the first, second and third postoperative year was performed by $t$-tests.
*Results:* Radiographic analysis reveals two patterns, variably mixed in each case: (A) when MBA cracks, load over VFA rapidly increases. Living fibula produces a fast, dense cortical hypertrophy. (B) MBA preserves its original strength and absorbs the majority of load. Living VFA presents a progressive but slower enlargement of both external and medullary diameter with a low-density cortex. Serial CT analysis demonstrated statistically significant changes in both VFA diameters ($p$<0.001) and areas ($p$=0.0001). These increments increased between the second and third p.o. year when all patients abandoned braces. From the second p.o. year, a layer of ossification appeared on the endosteal surface of the allograft; tissue between VFA and inner MBA showed a slow maturation with axial bridges between the two bones. After 5 years the inner fibula completely fused to the allograft that, if mechanically intact, appeared almost completely substituted by new reactive bone. VFA can rapidly adapt to new mechanical situations: cortical changes are directly correlated to load. Serial radiological analysis shows complete remodelling of MBA with inlaid VFA and suggests bone-inductive activity of VFA on the endostal surface of MBA.

**Results after five or more years of 95 allografts used in the treatment of mbt of children and adolescents**

M. San-Julian & J Ca adell
*(Dpto. Traumatología, Clínica Universitaria, Avda. Pio XII 36, 31008 Pamplona, Spain)*

*Aim:* To evaluate the long-term follow-up results of allografts used in limb reconstructions of children and adolescents.
*Methods:* 95 massive allografts used in 80 patients younger than 20 years were followed during 5 or more years (June 1987–January 1995). The mean age was 14 (4–19) years. The mean follow up was nine years (5-13). Forty-two were intercalary allografts, 19 were osteoarticular and 34 were composite allograft–prosthesis. The histological diagnoses were osteosarcoma (66 cases) Ewing's (11 cases), fibrosarcoma (three cases). All patients received chemo- and/or radiotherapy according to our protocols.
*Results:* 14 patients required two allografts due to complications or bone metastases of their primary sarcoma. Seven patients suffered infection, three suffered fractures and two required exchange of the allograft due to non-union. Functional results were better in intercalary allografts (88% of excellent and good results, including those who were treated for any complication). The number of patients with any complication was lower than the number of allografts having complications (some patients had two or more complications). Limb length discrepancies, when present, were successfully corrected by bone lengthening or taking benefit from any complication requiring allograft exchange.
*Conclusion:* Allografts in children and adolescent have a higher complication rate than those used in adults. However, this number of complications can be reduced very much by coverture of the allograft, using intramedullary nailing, advising the possibilities of late infection, etc.

**Tumours of the pelvis and hip joint: methods and outcome of surgical treatment**

J. Somville, S. Maiya, A. Taminiau & R. Grimer
*(Orthopaedic Oncology Service, Royal Orthopaedic Hospital, Birmingham, UK)*

*Purpose:* To assess outcome and function following surgery for non-metastatic primary bone tumours of the pelvis and the hip in order to try and ascertain the 'best' procedures for different tumour sites.
*Method:* 300 patients with non-metastatic primary bone sarcomas of the pelvis and proximal femur from three treatment centres in United Kingdom, Belgium and The Netherlands, have been reviewed to investigate the outcome both in terms of function, complications and survival. Standard assessment tools (MSTS and TESS scores) have been used.
*Results:* The tumours tended to present in similar ways—non-specific pelvic pain and referred pain—with long delays in diagnosis being common (mean 56 weeks). Tumour size averaged 13 cm at diagnosis and was little different for the three main tumours. There were 57 osteosarcomas, 49 Ewings, 185 chondrosarcomas and nine others. Tumour locations included 55% in the pelvis and 45% in the proximal femur. The distribution of the pelvic tumours were almost equal in the three sites (P Class) with a slight predominance in the acetabular area. Treatment for all tumours was surgical with appropriate adjuvant chemotherapy and or radiotherapy as indicated. The overall survival of the patients was 64% at 5 years and was related to tumour type and grade; 89% patients had limb salvage surgery and 11% had amputations. The survival rates following amputation were worse than after limb salvage. Prognostic factors for survival were assessed and will be discussed. Local recurrence arose in 24% patients and was related to: diagnosis, size, localization and surgical margins. Complications following surgery were related to type of surgery and margins. There were more complications following limb salvage surgery, with 6% patients subsequently requiring amputation because of failure of their limb salvage either related to local recurrence (23% cases) or major complications. Functional outcomes were worse in all patients with pelvic tumours than proximal femoral tumours but patients with uncomplicated Limb salvage did best.
*Conclusion:* Pelvic and hip tumours present the greatest challenge to orthopaedic oncologists. Successful Limb salvage surgery is always better than ablative surgery, but marginal surgery for sarcomas and inadequate reconstruction lead to both oncological and reconstructive complications which may mean that the patient is worse off than with a primary amputation. Careful assessment of all the risks must be taken into account when planning these procedures.

**Uncemented fixation of massive implants. the first 9 years**

J. Cobb, F. Muir, G. Blunn, J. Witt, T. Briggs & S. Cannon
*(The London Bone and Soft Tissue Tumour Service, The Middlesex Hospital and University College London Hospitals NHS Trust)*

Massive replacement for malignant disease has been our standard form of reconstruction. In the paediatric and adolescent skeleton, cemented fixation was successful in the short term, but inevitably failed in the medium and longer term. We developed uncemented fixation using hydroxyapatite coating in 1991.
*Method:* Patients with an osteosarcoma or other sarcoma were offered uncemented fixation as an alternative to cemented fixation if the resection plane had a reasonable length of diaphyseal bone remaining. One hundred and forty-six patients had massive replacements between 1991 and 1999. All had hydroxyapatite-coated stems and collars. They have been followed by serial radiographs and clinical signs.
*Results:* Eight prostheses(6%) have required revision for loosening. In all of these, primary fixation failed. All have been revised without incident. Eight have become infected (6%). three of these had documented Hickman line infections. Seven have been successfully revised with two stage procedures, while one was amputated. There were also seven amputations for local recurrence, and five other unplanned operations. Radiographs viewed serially show no progression of radiolucency in the well fixed prostheses, and a steady increase in the volume of bone at the shoulder of the prosthesis while the pedicle matures into the collar. Following this, the bone mass diminishes slightly, but in otherwise healthy patients, the bone mass and the bone-implant interface stabilise and do not deteriorate.
*Discussion:* Fixation with hydroxyapatite-coated stems in diaphyseal bone is reliable and shows no sign of deteriorating with age. Comparison with cemented implants shows that the uncemented interface is more reliable and durable. The interface matures with time and may represent real osseomechanical integration.

**Van Nes rotation plasty for malignant bone tumors of the distal femur**

A.H.M. Taminiau
*(Leiden University Medical Centre, Oncologic Orthopaedics, The Netherlands)*

*Introduction:* Limb salvage surgery for primary malignant bone tumors has become worldwide accepted. The development of new imaging techniques (MRI), neoadjuvant chemotherapy protocols and surgical reconstructive procedures (tumor-endoprosthesis, allografts) have made limb salvage feasible in the majority of the cases. However due to age, site, size and tumor extension, ablative surgery is inevitable in a certain amount of the patients. Curative treatment in combination with preservation of a substantial part of the leg and foot for tumors around the knee has recently been realised by the introduction of rotation plasty (van Nes) of the lower leg as an alternative for above knee amputation in those cases where other types of reconstruction are not feasible. In rotation plasty the tibia is rotated 180° degrees and fixed to the proximal femur. The ankle joint is planned at the same level as the opposite knee and can act functionally as a knee joint. The appearance of the rotated foot at the level of the knee compensated wearing the prosthesis still remains to be a cosmetic disadvantage. With this innovative and advanced surgical technique a wide resection of a malignant tumor is performed. A major advantage of rotation plasty is that postoperative rehabilitation of these patients can be established relatively early after the operation and, more importantly, that rehabilitation is superior to that after amputation through the upper thigh.
*Material and methods:* Our indications for rotation plasty are sarcoma of the distal femur (Enneking's Stage IIB), no involvement of the sciatic nerve, normal ankle function and acceptation of the procedure by the patients and their relatives. In 273 limb salvage procedures of primary malignant bone tumors of the femur 41 rotation plasties were performed in Leiden since 1982. The predominant diagnosis was osteosarcoma 33, Ewing five, MFH three and chondrosarcoma one. There were 22 men and 20 women. Medium age was 5 (two), 10 (four), 13 (six), 18 (16), 24 (three), 28 (three), 31 (one), 37 (five), 42 (one). We had stage IIB in 33 and stage IIIB in nine. The main contraindication is tumor involvement of the sciatic nerve. There were no recurrences. Twenty-four patients died of their disease due to metastases: <1 year, nine;

1–2 years, eight; 2–3 years, six; and after 4 years, one. Eighteen are alive without evidence of disease, including two after metastasectomy >5 years after thoracotomy. Detailed quality of life in the surviving group of patients was studied. The results show that physical functioning was poorer than healthy persons but psychological functioning was highly comparable. To get better insight in the feasibility of this procedure several items concerning technique and outcome were studied. This study encloses vascular resection and reconstruction instead of preservation, explanation for the absence of oedema, analysis of the muscle function after rotation and the functional results.

*Discussion:* Rotation plasty of the lower leg is a functionally attractive alternative to conventional amputation through the upper thigh for malignant tumors of the distal part of the femur. Rotation plasty in many instances provides wider surgical margins than traditional limb salving resections while providing better function then traditional amputation. However, the prognosis is poor in this group mainly due to the large tumours requiring this alternative for amputation.

**References**

1 Taminiau AHM, Van der Eijken JW, Van Bockel HJ, Sobotka MR, Obermann WR, Taconis WK. Role of vascular resection and reconstruction in rotationplasty. In: T.Yamamuro, ed. *New Developments for Limb Salvage in Musculoskeletal Tumors.* Tokyo: Springer, 1989: 133–5; ISBN 4-431-70030-70.

2 Taminiau AHM, Arndt JW, Bockel JH van. Evaluation of the lymph flow with lymphoscintigraphy after rotation plasty for the treatment of bone tumors. J Bone Joint Surg 1992; 74A: 101–5.

3 Steenhof JRM, Daanen HAM, Taminiau AHM. Functional analysis of patients who have had a modified Van Nes rotation plasty. J Bone Joint Surg 1993; 75A: 1451-1456.

4 Veenstra KM, Eyken JW van, Taminiau AHM. Quality of life in survivors with a Van Nes rotation plasty after tumor resection. in press.

**Function after 'hindquarter amputation'**

J. Metcalfe, R.J. Grimer & C Eiser
*(The Royal Orthopaedic Hospital Oncology Service, Bristol Road South, Birmingham, UK)*

*Purpose:* Hindquarter amputation was once described by Gordon Gordon-Taylor as '… a rather vulgar term but one which expresses the extent of the human immolation'. Despite several papers describing the outcome of hindquarter amputation in terms of patient survival and complications, there has been no structured review of survivors to assess their quality of life and functional outcome.

*Material and method:* We have performed 103 hindquarter amputations in the past 28 years, mostly for chondrosarcomas of the pelvis but also for osteosarcoma, radiation-induced sarcoma and soft tissue sarcomas. Twenty-one of the patients remain alive and disease free and agreed to participate in answering a questionnaire which had been accepted by the local Ethical Committee. We used well accepted tools to measure quality of life (SF 36), function (MSTS and TESS scores), sexual functioning (IIEF) and we also asked specific questions about the use of artificial limbs and the problems encountered.

*Results:* The SF36 scores confirmed the generally low level of achievement of all patients, well below the norm but interestingly, when compared with other patients with long standing illness they had comparable scores for social functioning, mental health and general health perception. Wound healing took an average of 5.7 months. All had been offered an artificial limb but only four regularly wore it for most of the day. Nine never used the artificial limb. The main complaints were that it was heavy and cumbersome and had to be taken off completely for defecating (all patients) and for passing urine (females). Phantom pain was very common, still being experienced in all but one patient, treatment was on the whole unsuccessful. The TESS scores revealed many difficulties in daily life, especially gardening, kneeling, walking up hills and self rating. The IESS score confirmed that impotence was the norm following these operations, causing considerable distress to those concerned.

*Conclusion:* Hindquarter amputation is usually only considered as a last resort in terms of controlling cancer. The information obtained from this study documents the true price patients pay for undergoing this operation.

**Non-surgical local treatment for high-grade non-metastatic osteosarcoma (OS) of the extremities**

G. Matchak, P. Sinyukov, S. Tkachev, V. Tepliakov, B. Bokhian & A. Ryjkov
*(N.N. Blokhin Cancer Research Centre, Kashirskoye sh.24, 115478 Moscow, Russia)*

*Purpose:* To evaluate the oncological outcome of patients, that were treated with neoadjuvant chemotherapy and who refused surgery for primary tumour.

*Patients and methods:* Between January 1, 1988, and December 31, 1999, 23 OS patients received primary chemotherapy (PCH), local radiotherapy 55–60 Gy ± adjuvant chemotherapy. The ages ranged from 14 to 30 years with a mean of 18. There were 11M/12F. The most common sites were proximal tibia (16) and distal femur (five). PCH consisted of three to five cycles of intra-arterial CDP 150 mg/m$^2$ (17 patients) or DOX 90 mg/m$^2$ (six patients). During induction, the imaging response was assessed by analysis of tumour size, sclerotic changes of extraosseous mass (X-ray, CT, MRI), tumour neovascularisation and perfusion (angiography, three-phase bone scintigraphy), $^{99m}$Tc uptake (plain scintigraphy) and alkaline phosphatase levels. When all factors were evaluated as effective the final evaluation of the imaging response was effective. Limb function was estimated according to the Enneking classification.

*Results:* Long-term follow-up (median 39) has shown an overall 5-year survival of 52% (95% CI 25–78%). Fourteen patients are continuously progression-free 6–94 months (median 36). Limb function after PCH and during follow-up was excellent in 19 patients (83%). Nine patients relapsed, four with metastatic disease, one with local progression (LP) and four with both. Local progression occurred in five (22%) cases, from 5 to 48 months (median 15) after the completion of radiotherapy. Eight patients (35%) developed metastases from 5 to 65 months (median 10). Prognosis was closely related to the chemotherapy-induced imaging response. Retrospectively, 10 of 23 patients (43%) were classified as imaging responders (IR), 13 as non-responders (NR). No LP were observed in IR compared with five LP in NR ($p$<0.02). Two of five patients with LP were treated by amputation and one by bone resection with endoprosthetic replacement. The 5-year actuarial metastases-free survival rate was 100% (95% CI 100–100%) and 36% (95% CI 11–73%), respectively, for IR and NR ($p$<0.001). Two pathological fractures and three postradiation fibrosis were noted after radiotherapy.

*Conclusions:* In patients with pronounced imaging response to PCH, the substitution of surgery by radiotherapy in a dose 55–60 Gy had no adverse effect on the local and systemic disease control. Perhaps, a new approaches for local OS treatment will be discussed in the near future.

**Minimally invasive radiofrequency therapy for the treatment of local tumour recurrence**

M.A. Hall-Craggs, J.D. Witt, J.P. Cobb, S. Bown, J. Whelan & A. Cassoni

*Introduction:* Surgical resection with wide margins offers the best chance of achieving a cure and preventing local disease recurrence in patients with low grade malignant and aggressive benign musculoskeletal tumours. If disease recurs treatment options are restricted as surgery may be limited by the tumour site and extent, and these tumours are relatively chemo- and radio-insensitive. An alternative approach to controlling local disease is to use locally applied minimally invasive therapy and a number of different techniques are currently being investigated. These aim to cause maximum local tumour destruction with minimal collateral damage. In this pilot study we report our initial experience of using radiofrequency thermal ablation in five patients with local tumour recurrence where disease was not amenable to conventional therapy.
*Methods:* Three women and two men with locally recurrent tumours (three chondrosarcomas, one chordoma and one MFH) were treated with percutaneously applied radiofrequency probes. The RF systems (Radionics, Europe) were of a cooled tip design and single probes or clusters were used. Probes were positioned using CT, MR or ultrasound guidance and all treatment was performed as an inpatient procedure under general anaesthesia.
*Results:* Two patients had single treatments and the remainder had two or more treatments. Therapy in the two patients with large volume disease was not clinically effective in the short to medium term, although there was treatment effect, this was insufficient to control the growing disease. Two patients with small volume disease (<20 ml) currently have good local disease control at 18 and 11 months follow-up. The remaining patient opted for further surgery shortly after the RF therapy. Complications of the technique include skin necrosis (one), local discharge (two), bowel fistula (one) and transient nerve damage (two).
*Conclusions:* These preliminary results suggest that local disease control may be gained in patients with small volume tumour recurrence but that previous surgery and radiotherapy may increase the likelihood of treatment complications.

**Stump lengthening after hip disarticulation by using a Mutars® endoprosthesis**

G. Gosheger, A. Hillmann, N. Lindner, T. Ozaki & W. Winkelmann
*(Department of Orthopaedics, University Hospital, Albert-Schweitzer-Str. 33, 48149 Muenster, Germany)*

*Introduction:* A severe functional and cosmetic loss develops after hip disarticulation to obtain wide surgical margins. In contrast to hip disarticulation, above-knee amputation is much more common after trauma or ischemia, and people are familiar with the functional and cosmetic aspect.
*Methods:* We developed a special surgical procedure in patients with tumors of the proximal femur involving the sciatic nerve. After hip disarticulation a flap is preserved. A modular endoprosthesis is placed into the acetabulum. A trevira tube is applied to the reconstruction of the joint capsule and fixation of soft tissues. We perfomed this procedure in five patients. One patient had an infected endoprosthesis, one a malignant fibrous histiocytoma, one a fibrosarcoma, one a leiomyosarcoma, and one a hemangioendothelioma. The follow-up ranged from 3 months to 20 months (3, 6, 19, 19, 20 months).
*Results:* The primary wound healing did not present any problems. We could reach stump lengths of 23, 27, 31, 25 and 41 cm. All patients could wear an above-knee prosthesis. According to the MSTS-score (Enneking), the functional results were good. We did not observe severe blisters or horny skin after wearing the prosthesis especially in load bearing zone. All patients could walk without crutches for short distances. For ambulation of middle and longer distances the patients used one crutch or a cane. The radiographic follow-up showed good articular conditions and no local recurrence. One patient died because of pulmonary metastasis.
*Conclusion:* Stump lengthening by using a modular prosthetic system is a convincing alternative to hip disarticulation. The short-term results show good functional results. The middle- and the long-term results have to be evaluated. Even for the upper extremity this possibility of reconstruction can be available.

**Evaluation of chemotherapy response in primary bone tumors with F-18-FDG-PET in comparison to TC-99m-MDP bone scintigraphy**

F. Böttner, C. Franzius, N. Lindner, J. Sciuk, C. Brinkschmidt, H. Jürgens & W. Winkelmann
*(Departments of Orthopaedic Surgery, Nuclear Medicine, Pathology and Pediatric Oncology, University of Muenster, Germany)*

*Purpose:* Usually, bone scintigraphy is used for the assessment of neoadjuvant chemotherapy response of osteosarcomas and Ewing's sarcomas. We evaluated the potential of F-18-FDG-PET to assess chemotherapy response.
*Patients and Methods:* 15 patients with primary osseous tumors (10 osteosarcomas, five Ewing's sarcomas, age range 5–36 years, median 13 years) were studied with F-18-FDG-PET and planar Tc-99m-MDP bone scintigraphy before neoadjuvant chemotherapy and preoperative. Tumor response was classified histologically by analysis of the resected specimen according to Salzer-Kuntschik (grade I–III, good response with <10% vital tumor areas; grade IV–VI, poor response with Š10% vital tumor areas). PET was performed in whole-body technique (scanner ECAT EXACT 47, Siemens), bone scintigraphy in usual three-phase technique. In both imaging modalities, quantification was done using tumor/non-tumor ratios (T/NT). The percental reduction in T/NT ratio between baseline and follow-up examination was determined.
*Results:* 13 patients were classified as good responders (six patients II, seven III), two patients were classified as poor responders (IV and V). FDG-PET showed a more than 30% decrease in T/NT ratios in all good responders, whereas three of these patients had increasing bone scan T/NT ratios, and another four had decreasing ratios of less than 30%. In one of the two poor responders FDG-PET T/NT ratio was enhanced, and bone scan T/NT ratio showed a low reduction of 19%. The other poor responder had a decreasing FDG-PET T/NT ratio of 11%, and an increasing bone scan T/NT ratio.
*Conclusion:* The low correlation between PET and bone scanning indicates that different aspects of the tumor metabolism are measured. In this small patient group, F-18-FDG-PET accurately assesses the effect of chemotherapy on primary osseous tumors.

**Poster presentations**

**Neoadjuvant chemotherapy cisplatin (DDP)+adriamicin (ADR) combined with regional hyperthermia (RHT) in the treatment of primary high-risk soft tissue sarcomas**

B. Bokhyan, S. Ivanov, R. Karapetyan, A. Gevorkian., V. Tepliakov & S. Tkachov
*(N.N. Blokhin Russian Cancer Research Centre., Kashirskoe sh. 24, Moscow 115478, Russian Federation)*

*Aim:* The efficacy of neoadjuvant thermochemotherapy was investigated.
*Patients and methods.* We report the results of phase I/II studies of treatment of 22 patients with II and III grade extremities soft tissue sarcoma (STS). All patients had extracompartmental lesions, tumour size > 8 cm (mean 12 cm). Mean tumour volume was 540 $cm^3$. The preoperative chemotherapy with DDP 120 $mg/m^2$ and ADR 90 $mg/m^2$ (1 day) for 6 weeks (two cycles) combined with two fractions of RHT (60 min, 43.0°C) days 1, 3.
*Results:* Limb-saving surgery was performed in 19 (86.4%) cases consisting of wide compartmental excision of the tumour. Mutilating surgery was performed in three cases. Treatment efficacy was assessed by clinical, morphological response and follow-up for systemic and local relapse. The efficacy rate was 50% or more. The mean tumour necrosis (>70% cells) rate in the resected specimens was 81.8%. There was no correlation between the histological response and the observed reduction in tumour volume. Postoperative complications were observed in 10 (45.5%) patients; among these, four patients developed wound infection that required surgical treatment as a complication of surgery performed in the early stage following the preoperative treatment. After a mean postoperative follow-up of 27 months, distant metastasis occurred in six (27.3%) patients resulting in five fatalities. The 3-year cumulative survival rate was 64.3%. No local recurrence was observed in any patient during the follow-up, thus confirming our hypothesis that DDP+ADR+RHT treatment has an excellent local efficacy.
*Conclusions:* The results of this study suggested that DDP+ADR+RHT was an effective local treatment for limb salvage in limb-threatening STS. We think that it would be valuable to conduct, at many facilities, phase III studies on the treatment of soft tissue sarcoma by a combination of surgery and preoperative multidisciplinary treatment using hyperthermia.

**Cytological diagnosis of high-grade osteosarcoma**
O. Brosjö, A. Kreicbergs, H.C.F. Bauer, V. Söderlund, O. Larsson & L. Skoog
*(Orthopedic Oncology Service, Karolinska Hospital, Stockholm, Sweden)*

*Patients and methods:* In a consecutive series of 47 high-grade osteosarcoma (OS) cases 1992–99, fine needle aspiration biopsy (FNAB) was performed in all.
*Results:* According to age, location, and radiological appearance, 31 of the 47 lesions were typical, classical, high-grade OS. FNAB was diagnostic in 30 of these 31 cases. In one of four cases undergoing also open biopsy, cytology showed sarcoma NOS, whereas histopathology classified the lesion as high-grade OS. As for the remaining 16 cases, eight had an atypical radiological appearance. In these, cytology was diagnostic for high-grade OS in three cases. The remaining five were cytologically classified as sarcoma NOS (two), low-grade OS (one), GCT (one), and inconclusive (one). In another subset of eight cases, both location and radiology were atypical. Cytology was diagnostic for high-grade OS in two cases. The other six were classified as sarcoma NOS (three), Ewing's sarcoma (one), and inconclusive (two). Because of inconclusive cytology, non-specific sarcoma or incongruity between clinical data, cytological diagnosis, and/or radiological appearance, open biopsy was done in 14 of these 16 cases. Thus, in two cases, definitive surgery was done without previous open biopsy as FNAB with certainty classified the lesions as low-grade OS and Ewing's: (1) a low-grade OS according to cytology (78 years old) was reclassified after wide excision as a small cell OS; (2) a Ewing's sarcoma in distal radius (14-year-old boy) with clinical, radiographical and immunohistochemical findings complying with this diagnosis, was treated preoperatively according to our Ewing's sarcoma protocol followed by wide local excision (NED after 5 years follow-up). However, histopathological examination of the surgical specimen disclosed an extremely uncommon small cell OS type, i.e., the epithelioid variant, which is reported to exhibit not only a similar histological picture as seen in Ewing's/PNET, but also the same cytogenetic translocation. Among the 16 atypical cases, histological assessment was associated with diagnostic difficulties in seven, requiring second opinion by international expertise.
*Conclusions:* Our study shows that FNAB is reliable as a confirmatory diagnostic tool in typical, classical, high-grade OS. Thus, in two-thirds of the cases open biopsy can be obviated. However, in atypical cases, which are easily recognised, open biopsy should be employed.

**Results of the limb-preserving reconstructions in malignant or metastatic tumours**
A.N. Makhson & A.S. Burlakov
*(Moscow Oncological Hospital #62, Moscow, Russia)*

At the present time 324 patients who were operated for malignant tumours of extremities by means of different reconstructive methods have been observed in our hospital during 5–28 years. Revascularized fibular bone graft, different free soft tissues flaps and endoprotheses were used in extremity restorations. Microsurgical procedures were performed successfully in 88 reconstructions. None of the other surgical techniques allows to replace extensive soft tissues defects. The free fibular bone grafting (32) is a method of choice in cases of diaphyseal lesions. We did not have any serious complications except bone graft fracture (four) which happened in 1–2 years after the operation. The use of endoprotheses is the most widespread and routine procedure. We used it in 236 cases: 149 proximal part of femur, 76 distal part and 11 total femur prostheses. The most serious complications that we had were among the distal part femur reconstructions and appeared in 15–28 years after operation (seven). In six cases we have to remove the endoprosthesis and repeat reconstruction using free fibular bone grafting. In one patient we used Van Ness procedure. It is a really serious problem to get bone consolidation in such patients. Metallosis and dislocation of endoprosthesis that happens during this period becomes a very serious problem, and can lead to amputation of the saved extremity. Based on our experience in treatment of such patients we can conclude that in cases of distal lesion of the femur and good prognosis of survivability, bone graft microsurgical transferring should be managed despite creating knee arthrodesis, especially for young patients.

**Periosteal osteosarcoma**
C. Zambakidis, S.R. Cannon, T.W.R. Briggs, J. Cobb, A. Saiffudin & J. Pringle
*(Institute of Orthopaedics, RNOHT Stanmore; The Royal National Orthopaedic Hospital, Stanmore; The London Bone and Soft Tissue Sarcoma Service)*

Periosteal osteosarcoma is a rare variant of osteosarcoma. 16 cases presenting between 1980 and 1996 have been reviewed. There were 12 female patients and their age ranged between 9 and 72 years. The predominant site was the proximal tibia, followed by the distal femur. Many patients had a fairly prolonged period of symptomatology up to 36 months prior to surgery. Treatment was diverse varying from disarticulation to wide local excision with or without graft, occasionally prosthesis was used. The historical patients did not receive chemotherapy, 13 subsequent patients have been treated with standard osteosarcoma chemotherapy but often under shortened courses. The overall survival in patients treated by modern therapy is 92%. Results of reconstruction based on muscular tumour society scoring varies from 20 to 100% with a mean of 70%. There is some evidence that patients treated by modern chemotherapy and local resection do better than historical controls.

**Total femoral endoprosthetic replacement**

L.A. David, S.R. Cannon & T.W.R. Briggs
*(Royal National Orthopaedic Hospital Trust, London Bone and Soft Tissue Tumour Service)*

*Study aim:* A review of 11 patients who underwent total femoral excision and reconstruction for malignant tumours with assessment of outcome using the Musculoskeletal Tumour Society (MTS) Rating Score.
*Methods:* Information was gathered from Database records, patients' case notes and by clinical review. The prostheses were all custom-made incorporating either a Stanmore Mk-5 knee mechanism or a SMILES (Stanmore Modular Individualised Lower Extremity System) device with four of the prostheses being extendable for use in children.
*Sample:* All patients who underwent total femoral replacement for malignant bone tumours at the RNOH between June 1978 and August 1999 were included. Of the 11 patients, six were male and five were female. The age at operation ranged from 5 to 65 years, with a mean age of 26 years and 8 months. The tumour type was osteosarcoma in seven cases, chondrosarcoma in two, Ewing's sarcoma in one and malignant fibrous histiocytoma in one. The patients had previously undergone a mean of 3.5 procedures (range 1–24). The mean follow-up was 39 months (range 7–111 months). Ten patients also received chemotherapy and three received radiotherapy.
*Results:* 2 patients died of metastases within 1 year. Three more died within 5 years. Seven patients developed metastatic disease. Three patients developed recurrences, which were treated with excision and radiotherapy. Two patients needed revision of the whole prosthesis (one for infection and one for failure of the growing mechanism) and one patient needed revision of the acetabular component due to dislocation. Of those who survived 1 year or more the average range of motion was 90° at the hip and 80° at the knee. Using the MTS rating the outcome of one patient was excellent, five were good and three were fair.
*Conclusion:* Total femoral endoprosthetic replacement can be used effectively in patients after excision of malignant tumours in order to preserve the limb, with good function achieved in some cases. However, there are many risks involved and the outcome is extremely varied.

**Oncological outcome aftfr intralesional surgery in chondrosarcoma**

T. Charatishvili, Yu. Soloviev, M. Aliev & N. Trapeznikov
*(N.N. Blokhin Cancer Research Centre, Kashirskoye sh. 24, Moscow, 115478, Russian Federation)*

*Purpose:* To evaluate the outcome of 94 patients who underwent IL surgery for chondrosarcoma.
*Methods:* There were 61M/33F. The ages ranged from 16 to 70 years. with a median of 40. Fifty-four tumours were located in the axial skeleton and 40 in the extremities. The histological grade distribution of the chondrosarcoma was: 23 Grade I, 48 Grade II, 13 Grade III, five mesenchymal and five dedifferentiated. Median follow-up time was 99 months.
*Results:* The 10-year overall survival rate for the 94 patients who underwent IL surgery was 37%, and 56% for the 227 patients who underwent adequate surgery ($p$=0.02). The incidence of local recurrence was 79 cases (84%) after IL surgery compared with 53 (23%) after radical surgery ($p$<0.0001). The 10-year metastases-free survival rate for the who underwent IL surgery was 33%, and 54% for patients who underwent wide or marginal surgery ($p$<0.002). In patients with IL surgery, the overall survival rates for the patients with tumours of histological Grade I, II and III+ mesenchymal+dedifferentiated was 73, 33 and 12%, respectively ($p$<0.0001). The overall survival rates for patients with central tumours and extremity tumours were 29 and 47%, respectively ($p$>0.05).
*Conclusions:* IL surgery for chondrosarcoma often leads to local relapse that results in metastases and death. The prognosis for patients after IL surgery tends to differ according to tumour grade.

**Joint sparing surgery for malignant disease**

J. Cobb, J. Witt, G. Blunn & P. Unwin
*(The London Bone and Soft Tissue Tumour Service, The Middlesex Hospital and University College London Hospitals NHS Trust)*

Massive replacement to reconstruct after resection of malignant tumours is difficult close to joints. Often the joint is sacrificed despite being normal because implant fixation close to the joint has been impossible.
*Method:* Extracortical plates (ECP) have been used in 27 cases for metaphyseal fixation where conventional intramedullary stem (IMS) fixation would have been impossible.
*Results:* At a mean of 22 months, no loosening has been observed, and normal joint function has been retained. Prominent screw heads have caused some minor discomfort.
*Discussion:* ECP fixation appears a safe mechanical alternative either with a biological or massive reconstruction. The wide metaphyseal bone surface accommodates rapidly to the fixation with no obvious loss of joint function. We now consider that 10 mm of subchondral bone either side of the knee is sufficient for joint salvage surgery. Routine resection of normal joints for reconstruction purposes for oncological clearance should be reduced by up to 40%.

**Adamantinoma of the long bones—the first bilateral case**

M. Dominkus[1], I. Sulzbacher[2], C. Oettl[3], R. Kotz[1]
*([1]Department of Orthopedics, [2]Institute of Clinical Pathology, and [3]Department of Radiology, Vienna Medical School, Waehringer Guertel 18–20, A-1090 Vienna, Austria)*

Since 1973, six patients with adamantinoma of the long bones were treated in our institution. All of the two men and four women with a mean age of 45.7 years (33.7–62.8) had their location in the tibia. The most recent case showed skip metastases and a simultaneous presentation on both tibias. The time of clinical symptoms ranged from 2 to 34 years. Most patients complained of pain and swelling in the diaphysis of the tibia or pathological fracture. Histological diagnosis after incisional biopsy revealed adamantinoma in all cases, with tubular or spindle cell pattern and epithelial nests which ruled out the differential diagnosis of osteofibrous dysplasia. The one patient with bilateral presentation was the only one with skip lesions. There was a single skip lesion in the left tibia proximal to the tumour and two skip lesions were found in the right tibia, one located proximal and one distal to the main mass. The histology of these lesions did not differentiate from the main tumour. The treatment consisted of marginal resection in two, curettage in one, amputation one and wide resection with consecutive biological reconstruction with vascularized fibula or fibula pro tibia in two patients (three legs). Local recurrence occurred in four patients. After a follow-up period of 4 months to 15 years, three patients showed no evidence of disease, one patient died of disease, one is alive with disease and one patient was lost to follow-up 1 month postoperatively. The presented case of a bilateral adamantinoma is the first case reported in the literature, so far. Skip lesions in one bone may promote a multifocal presentation.

**Malignant lymphoma of the bone**

H.R. Dürr, P.E. Müller, E. Hiller, A. Baur & H.J. Refior
*(Department of Orthopedics and Orthopedic Surgery, Ludwig-Maximilians-University Munich, Klinikum Grosshadern, 81366 Munich, Germany)*

*Study aim:* Malignant lymphoma of the bone is rare. In many cases, its diagnosis is delayed because of unspecific clinical signs and equivocal radiographs. Therapy in general is multimodal, including surgery and radio- and chemotherapy. Our objective was to demonstrate the clinical and radiological aspects of the lesion to optimize diagnostic approaches and to evaluate treatment and prognostic factors.
*Methods:* Thirty-six cases of malignant lymphoma of the bone that were surgically treated over a 15-year-period were retrospectively reviewed. Seventeen of the cases showed a singular bone non-Hodgkin's lymphoma (NHL) which classified them as primary lymphoma of the bone (PLB). In 13 cases, dissemination of the disease with multiple bone or visceral involvement was apparent. Six patients suffered from bone involvement due to Hodgkin's disease. Surgical treatment was indicated for diagnostic reasons or complications due to the disease. Radiation and chemotherapy were part of the oncological treatment.
*Results:* The mean age was 57 years. The main symptom in malignant bone lymphoma in 33 patients was pain, with an average duration of 8 months. In the secondary cases, bone involvement appeared on average 57 months after the initial diagnosis. Fifty-eight percent of the lesions showed an osteolytic pattern. Soft-tissue involvement was seen in 71% of the cases and was the primary diagnostic sign associated with this disease. The 5-year survival rate was 61%. Multiple versus solitary bone involvement was the most significant factor in the prognosis. Extraskeletal involvement significantly decreased survival. No correlation was found between gender, age, location or histological sub-types, and survival.
*Conclusions:* Bone involvement in NHL appears late in the extraskeletal disease. The clinical appearance is non-specific, and the delay between the onset of symptoms and diagnosis is often long. One of the major radiological signs is the existence of a soft-tissue tumor surrounding the bone with little or no bone involvement on plain films. Treatment generally, is nonsurgical, and based on the stage of the disease. Local radiation with or without systemic chemotherapy should be used. Long-term survival is favorable, but dependent on the stage of the disease and the amount of bone involvement.

**The Mutars®-trevira tube for reconstruction of capsule and soft tissue in the endoprosthetic replacement of bone tumors**

G. Gosheger, A. Hillmann, R. Roedl, Ch. Hoffmann & W. Winkelmann
*(Department of Orthopaedics, University Hospital, Albert-Schweitzer-Str. 33, 48149 Muenster, Germany)*

*Introduction:* Large defects after resection of a malignant bone tumor can be reconstructed by using a modular endoprosthesis. A special problem consists of replacing the soft tissues and joint capsules to the endoprosthetic device. There have been some reports on dislocation of megaendoprostheses especially in the proximal femur, total femur, and proximal humerus.
*Methods:* From 9/92 to 12/98 in the orthopedic department of the University of Muenster, 69 Mutars-megaprostheses were implanted using a trevira tube. The endoprostheses were implanted after proximal femur resection ($n$=33), total femur resection ($n$=5) and proximal humerus resection ($n$=16). In these cases the trevira tube is used for the reconstruction of the capsule and for the refixation of muscles, and so far for avoiding dislocation. In the case of proximal tibia replacement ($n$=7), arthrodesis of the knee joint ($n$=3), total knee ($n$=2) and distal femur replacement ($n$=3) the Mutars–trevira tube allows attaching muscle-flaps and extensor tendon. The minimal follow-up was 9 months, the maximal follow-up was 78 months, and the mean follow-up was 31.6 months.
*Results:* Regarding the unconstrained megaendoprostheses (proximal femur, total femur, proximal humerus) a dislcocation was observed only in two of 52 patients (3.8%). These two cases were proximal femur endoprostheses. No dislocation was observed in patients with a total femur endoprosthesis or a proximal humerus endoprosthesis. Futhermore the trevira tube was used to attach the gastrocnemius muscle in patients with a proximal tibia endoprosthesis and to reattach the rotator cuff in patients with a proximal humerus endoprosthesis. As for the infection rate, we could not observe significant increase of infection rates ($p$=0.46). The histopathological findings of six revision cases showed a complete tissue ingrowth into the tube.
*Conclusion:* By using the Mutars®-trevira tube for reconstruction of capsule and soft tissue dislocation of megaprostheses can be reduced. Furthermore the trevira tube can serve for the attachment of flaps and muscles.

**Results of reconstructive surgery for shoulder tumours**

R. Gradinger; H. Rechl & R. Burgkart
*(Clinic of Orthopaedic Surgery, Technical University Munich, Ismaninger Str. 22, 81675 München, Germany)*

From 1971 to 1987, 85 patients with primary ($n$=45) and secondary ($n$=47) malignant bone tumours of the shoulder region were operated. The performed treatments were: only tumor resection ($n$=30), refixation of the remaining humerus to the clavicula or thoracic wall ($n$=4), cemented endoprotheses (Polyacetal, $n$=16; Carbon Fiber, $n$=8; Modular Metal, $n$=3), space holder ($n$=2), osteosynthesis augmented with methyl methacrylate ($n$= 14), fibula autografts (reconstruction of diaphysis ($n$=6) or proximal humerus ($n$=4)), one arthrodesis and three fore-quarter amputations. All resections of the primary tumors were 'wide' and of the metastases 'marginal' or 'intralesional'. The average age of the patients with endoprotheses was 52 years and with fibula autografts 21.9 years. The mean resection length was 12.7 cm (endoprostheses) vs. 19.6 cm (fibula). The functional *results* according to the ISOLS criteria of the endoprotheses were good for motion, strength, function and patient satisfaction and very good for pain, deformity and stability. Complications were luxations ($n$=2), local recurrences ($n$=2), loosening ($n$=1) and implant break ($n$=2). The functional results of the fibula autografts as diaphyseal spacer were good ($n$=3) or very good ($n$=3), but as reconstruction of the proximal humerus fair ($n$=3) or poor ($n$=1). As complications we observed fractures ($n$=3), which could be successfully treated by internal fixation.
*Discussion:* Most reconstructions of diaphyseal defects demonstrate excellent results, whereas salvage procedures of the proximal humerus with resection of the rotatory cuff lead to poorer functional results. An important difference between refixation of the remaining humerus to the clavicula or thoracic wall and the reconstruction of the proximal humerus with fibula or endoprothesis was not obvious. For the treatment of primary bone tumors we prefer biological solutions, whereas for palliative indications we mainly use space holders or modular endoprostheses.

**Chordoma of the sacrum and mobile spine. A study of 39 cases**

P. Bergh[1], B. Gunterberg[1], L.-G. Kindblom[2] & Jeanne Mesi-Kindblom[2]
*(Departments of [1]Orthopaedic Surgery and [2]Pathology, The Musculoskeletal Tumor Center, Sahlgren University Hospital, 413 45 Göteborg, Sweden)*

*Study:* A retrospective investigation of patients operated in Göteborg.
*Aim:* To identify prognostic factors. To demonstrate the importance of center-based treatment. To evaluate long-term outcome.
*Materials and methods:* Review and analysis of clinical and morphological characteristics, type and place of diagnostic procedures and surgery, and follow-up for 39 consecutive patients with chordoma.
*Results:* There were 30 sacral and nine spinal chordomas (3–20 cm, mean 8 cm) in 22 women and 17 men (mean age 55 years). The pre-operative diagnosis was based on fine needle aspiration (FNA) (16) or core needle or incisional biopsy (12) in those primarily treated at our center. Definitive surgical margins were wide in 23 and marginal or intralesional in 16. Mean follow-up was 8.1 years. Local recurrence occurred in 44%, metastasis in 28%. The estimated 10- and 20-year survivals were 64 and 52%. Local recurrence was significantly associated with metastasis and tumor-related death.
*Conclusions:* Larger tumor size, invasive diagnostic procedures outside our center, inadequate surgical margins, microscopic tumor necrosis, $K_I$=67>5% and local recurrence are adverse prognostic factors. FNA is the preferred method for histological diagnosis. *En-bloc* removal with adequate margins has improved local control and outcome, which did not differ between sacral and spinal tumor location.

**Talocrural joint contact stress in rotation plasty**

A. Hillmann[1], D. Rosenbaum[2], E. Eils[2], L. Renneberg[1] & W. Winkelmann[1]
*([1]Orthopaedic Department, Funktionsbereich Bewegungsanalytik (Movement Analysis Lab), and [2]University of Muenster, Albert-Schweitzer-Str. 33, 48149 Muenster, Germany)*

*Introduction:* After resection of a malignant bone tumor of the femur rotationplasty is considered to be a treatment option beside other limb salvage procedures like endoprosthetic replacement, allograft or autograft reconstruction. The shortened limb is adapted to a prosthetic foot with a customized shaft and can be fully loaded. However, due to the marked change in the loading conditions of the foot degenerative changes in the ankle joint may be promoted. The aim of the present study was to characterize the changes in the loading conditions in the talocrural joint in order to understand and—if possible—prevent the development of joint degeneration.
*Materials and methods:* Nine fresh-frozen leg specimens were measured in two conditions: in a simulated single-leg stance and in a simulated rotationplasty with the foot rotated and embedded in a cast that was connected to a prosthetic foot. A loading frame with a load cell above the specimens and a force platform underneath was used to apply a load of 600 N (estimated body weight) and to control the experimental conditions. The load was gradually increased to the desired force level, kept constant for 10 s and released. Fuji prescale film (type superlow) was cut according to the shape of the talocrural joint surface, sealed against moisture with surgical foil, and inserted in the joint between talus and tibia from an anterior approach. The stained areas on the Fuji film were scanned with a resolution of 300 dpi and analyzed with a freeware program (OSIRIS) that determined peak and mean color intensity (i.e., pressure), area and force. Furthermore, the distance of the contact area to the edge of the talar facet was measured.
*Discussion and conclusions:* The present results demonstrate that the foot that is loaded in an extreme equinus position after rotationplasty is subjected to a marked change in talocrural joint contact characteristics. Even though the externally applied load was controlled the joint contact force decreased. This is most likely due to the fact that the foot is embedded in a tightly fitted shaft that leads to a dissipation of a certain percentage of the force. The expected posterior translation on the talar dome lead to a decreased contact area. This was also associated with a higher contact stress that might predispose the patients to an accelerated joint degeneration. The options for modifications of the prosthetic design are limited but will be subject of further investigations.
*This project was supported by a grant from the German Cancer Society (Deutsche Krebshilfe e.V.). Grant No.: 70-2460-Hi I.*

**Chondrosarcoma of the pelvis, a 10-year review**

D.G. Houlihan-Burne, T.W. Briggs & S. Cannon
*(The Bone Tumour Unit, The Royal National Orthopaedic Hospital, Brockley Hill, Stanmore, Middlesex, UK)*

*Study:* A clinical, operative and histological review of 33 patients with chondrosarcoma of the pelvis seen at our unit over a 10-year period.
*Aim:* To examine the mortality rate and rate of recurrence with respect to grade and anatomical site of tumour. Functional outcome of patients depending on the type of surgical procedure performed was also studied.
*Methods:* A retrospective analysis of cases.
*Subjects:* All patients seen at our unit over a 10-year period with a primary diagnosis of chondrosarcoma of the pelvis.
*Results:* Of the patients seen, 28 had a primary tumour diagnosed, the remaining five had recurrent tumours. The tumours were situated in the acetabulum (14), the pubic/ischial rami (seven), the iliac wing (six), the sacroiliac joint (four) and one in the sciatic notch and in the gluteal soft tissues. Surgical procedures included wide local excision (17), wide local excision and total hip arthroplasty (two), hemipelvectomy and endoprosthetic replacement (12), hemipelvectomy and fibula strut autologous bone graft (one), partial sacrectomy and reconstruction (one), and hindquarter amputation (one). The recurrence rate in the follow-up period was 30% (10/33) and this was related to local tumour spillage at the time of surgery, histological grade, anatomical area of tumour and type of operative procedure performed. Other complications of these procedures included dislocation and infection of prosthesis requiring revision surgery.

**Hyperthermic isolated limb perfusion with TNF-A and melphalan in advanced soft tissue sarcomas: histopathological considerations.**

I.Y. Kollender, M. Gutman, Lev-Chelouche, S. Abu-Abid, Merimsky, I. Bickels, A. Nirkin, G. Flusser, N. Maruani, B. Lifschitz-Mercer, M. Inbar, J.M. Klausner & I. Melter

*Background, materials and methods:* The specimens of 27 high grade extensive soft tissue sarcomas (STS) and three desmoid tumors of the extremities, after local treatment with hyperthermic isolated limb perfusion (HILP) using TNF-a and melphalan, were evaluated for the type and extent of tumor necrosis and other histological local tissue changes. Limb preservation was the objective in this selected group of advanced STSs, candidates for amputation or mutilating surgery otherwise. The tumoral masses were obtained 6–8 weeks after HILP, during the definitive surgical resection of the residual tumor according to protocol.
*Results:* Typical histological changes were: cystic hemorrhagic necrosis in the center of the remaining tumor with pericystic extensive fibrosis. In eight cases more than 90% necrosis was achieved. In 14 cases the percent of necrosis was between 60 and 90% (including four cases of 80–90%). In eight cases less than 60% necrosis was obtained. No correlation was found between these histological responses and the anatomical location of the tumor, whether the tumor was primary or recurrent, the type of previous treatment (systemic chemotherapy, radiotherapy) and its size.

Some correlation was found with the histological type of tumor and with proximal or distal location in the limb.
*Conclusions:* This is the first serial histological description of the effect of high dose TNF-a and melphalan administered via HILP on the tumoral masses of limb STS. The small number of specimens and especially the variability of tumors precludes definitive conclusions from the observed correlations. Larger numbers and more homogeneity of the histological types are needed in future series.

**CT guided core needle biopsy for muscoloskeletal tumors-the diagnostic method of choice?**

G. Flusser, J. Issakov, N. Maruani, O. Merimsky, I. Meller, J. Bickels, A. Nirkin & Y. Kollender
*(Tel-Aviv Sourasky Medical Center, Tel-Aviv, Israel)*

Bone and soft tissue lesions, primary and secondary, benign and malignant were traditionally diagnosed mostly by open biopsies done at the operating room under general anesthesia. This procedure although very accurate from the diagnostic point of view has disadvantages by means of morbidity, tumor contamination and cost effectiveness. In the last decade with the better pathology techniques and more experience core needle biopsies are taking over in modern Orthopedic-Oncology centers.
*Materials and methods:* Between January 1998 and December 1999 114 CT guided core needle biopsies were performed in our institution. The same radiologist performed all biopsies. The same pathologist examined all the pathology material. There were 41 soft tissue lesions and 77 bony lesions. Age ranged from 20 to 85 years (mean 53 years). There were 51 females and 63 males.
*Results:* All biopsies were performed ambulatory with no immediate complications. In three patients a post biopsy hematoma was noticed, that resolved spontaneously. Of the bony lesions, 46 turned out to be malignant (nine high grade sarcomas, five lymphomas, seven multiple myelomas and 25 metastatic tumors). Eleven lesions turned out to be benign; in six cases the results revealed normal bone. In two cases the diagnosis was osteomyelitis. In 12 cases the biopsy result was inconclusive (15%) and open biopsy was necessary for diagnosis. Of the soft tissue lesions, 19 turned out to be malignant (16 soft tissue sarcomas, two lymphomas and one melanoma). Twelve lesions were diagnosed as benign tumors, three cases turned out to be normal tissue. Three cases (7%) were inconclusive and were followed by open biopsy. Two cases of soft tissue that were diagnosed as benign lesions on core needle biopsy turned out to be malignant at definitive surgery.
*Conclusions:* CT guided core needle biopsy for musculoskeletal tumors seems to be accurate and reliable. Although some improvement is required in our accuracy rate. We achieved overall accuracy rate of 87% (85% for bone lesions and 93% for soft tissue lesions). The complication rate is negligible and the cost effect is low. We recommend CT guided biopsy as the primary diagnostic approach for muscoloskeletal tumors.

**The cryo-hit system: a new device for cryosurgery of bones. characteristics and technique, illustrated by series of the first 40 cases**

I.Meller, J. Bickels, A. Nirkin & Y. Kollender
*(The National Unit of Orthopedic Oncology. Sourasky Medical Center, Tel-Aviv, Israel)*

*Introduction:* Traditionally, using cryosurgery for bones (the pouring of liquid nitrogen in an open system according to Marcov's technique) was rare because it is a cumbersome, uncontrolled and dangerous method with many complications. Israel's Galil-Medical Ltd developed a sophisticated, modular, and well controlled system for cryotherapy of tumors in soft tissues (liver, prostate). We describe its modification for application in bones using a special gel-medium and a specific set of probes developed for freezing bony walls.
*Materials and methods:* From 12/97 to 3/99, 40 patients (26 males and 14 females; age, 14–78 years) were treated with the new device.
*Diagnosis:* Giant cell tumor (GCT), 15; aneurysinal bone cyst (ABC), seven; chondromyxoid fibroma (CMF), one; chondroblastoma, two; chondrosarcoma grade I, seven; metastatic bone disease (MBD), seven; osteosarcoma (OS), one.
*Anatomical locations:* Clavicle, one; proximal humerus, five; proximal ulna, one; distal radius, two; pelvis (including sacrum), 11; proximal femur, three; distal femur, eight; proximal tibia, five; distal tibia, two; foot (toe; calcaneus), two.
*Results:* The follow-up time is short (12–26 months). There was one LR (in the sacrum) and two fractures (late; healed by conservative management). There was no wound infection, no skin lesions and no neuro-vascular damages.
*Conclusions:* We present this new cryotherapy system in order to show its unique technical advantages over the old traditional and fearsome way of pouring liquid nitrogen into bony holes.

**Beromun® (TNF-A) and melphalan for limb salvage in advanced limb neoplasms: a new standard of care for irresectable soft tissue sarcomas**

I. Meller[1], M. Gutman[2], D. Lev-Chelouche[2], J.M. Klausner[2], J. Isakov, O. Merimsky, G. Flusser, N. Marouani, J. Bickels[1] & Y. Kollender[1]
*([1]The National Unit of Orthopedic Oncology, [2]Department of Surgery B, Sourasky Medical Center, Tel Aviv Israel)*

*Background:* Beromun® (recombinant tumor necrosis factor—rTNF_) is a highly potential antineoplastic agent. However, since its systemic administration in humans resulted in a life-threatening septic shock-like syndrome, its use was abandoned These systemic side effects were eliminated when Beromun® was administered via isolated limb perfusion (ILP). Following its success in treating metastatic melanoma confined to the limb Beromun® is now being used in order to prevent amputation or mutilating surgery in patients suffering from irresectable limb soft tissue sarcoma (STS). Promising results have been achieved in 260 patients participating in four clinical trials currently underway worldwide. An overview of these results is presented, with special emphasis on the data from the Tel Aviv Medical Center.
*Methods:* During a 7-year period, 70 patients with high grade STS underwent 81 ILPs with high dose Beromun® (3–4 mg) and melphalan (1–1.5 mg/kg). There were 37 males and 33 females. The mean age was 56 years (range 14–80 years). Histological subtypes included malignant fibrous histiocytoma, synovial, liposarcoma, malignant schwannoma, desmoid, clear cell, epithelioid, rhabdomyosarcoma, leiomyosarcoma, and unclassifiable. Thirty-one patients presented with recurrent and 40 with very extensive primary tumors. The tumors were located in the upper extremity in 13 patients and in the lower extremity in 57 patients. All patients were candidates for either amputation or extensive mutilating surgery. ILP was performed via the corresponding vessels proximal to the tumor. Resection of the residual tumor or tumor bed or limb was performed 6–8 weeks after ILP, and all reported responses were pathologically confirmed.
*Results:* Marked tumor softening occurred within 48 h, and in tumors protruding through the skin, hemorrhagic necrosis was evident within 24 h. The overall response rate was 80%. Twenty-one patients (30%) had a complete response and 40 (57%) had a PR. In three patients (11%), only minimal regression was observed

(stabilization of disease). Operative mortality was 3% (two patients). Limb sparing was achieved in 82% (62/70 patients). Amputation was performed in eight patients. Within a follow-up period of 2–76 months (median 24 months), 30 patients (43%) are dead, 31 (44%) are alive with no evidence of disease (median 49 months), and nine (13%) are alive with disease. Local occurrence occurred in 16/60 evaluable patients (26%). Limb salvage and survival rates were not significantly different for gender, age, primary vs. recurrent tumor, or tumor location. Mortality was significantly higher for multifocal disease (73 vs. 29%) ($p<0.05$). Limb salvage rates were also not different for the various histological subtypes. There was trend towards higher response rates in synovial and clear cell sarcomas.
*Conclusions:* The combination of Beromun® and melphalan given via ILP appears to be effective in patients with advanced STS confined to the limb, achieving a high response rate and limb preservation.

**Deep soft-tissue sarcomas of the extremities. management analysis of 120 consecutive patients**

J. Bickels, Y. Kollender, O. Merimski, J, Isakov, G. Flusser, A. Nirkin & I. Meller
*(The national Unit of Orthopedic Oncology, Tel-Aviv Sourasky Medical Center, Tel-Aviv, Israel)*

Deep soft-tissue sarcomas of the extremities often present as a large mass near the major neurovascular bundle of the extremity. The current study, based on the experience with 120 patients who were treated for deep soft-tissue sarcoma of the extremities, emphasizes the low local recurrence rate achieved by the judgmental use of neoadjuvant and adjuvant treatment modalities and optimal surgical margins.
*Materials and methods:* Between 1992 and 1995, 120 patients were diagnosed with deep soft-tissue sarcomas of the extremities. There were 67 males and 53 females whose age ranged from 2 to 90 years (median, 56 years), and who presented with 96 tumors of the lower extremity and 24 of the upper extremity. According to the ISOLS (international Society of Limb-Sparing) guidelines, 68% percent of tumors were graded as grade 4, 21% as grade 3, 7% as grade 2, and 4% as grade 1. Fifty-three percent of these tumors were immediately adjacent to the major neurovascular bundle of the extremity. Extremely large tumors or those in close proximity to the neurovascular bundle were treated with neoadjuvant chemotherapy or isolated limb perfusion (ILP) with TNF and melphalan. Wide surgical excision was attempted in all cases. Adjuvant radiation therapy was given when resection was performed close to the neurovascular bundle or when marginal resection (margins of less than 1 mm) or less were performed. Adjuvant chemotherapy was given to all patients who received neoadjuvant chemotherapy or to those patients who presented with large tumor prior to the introduction of the neoadjuvant protocol in 1995.
*Results:* Adjuvant chemotherapy was given to 22 patients and ILP was executed in 24 patients. Surgery achieved wide margins in 78 patients, marginal margins in 28, and radical margins in 11, in seven of whom by an amputation. Adjuvant chemotherapy was given to 23 patients and radiation therapy to 77 patients. All patients were followed for at least 2 years (range; 27–97 months). Sixteen patients (13.3%) had a local recurrence, occurring 3–30 months (median, 12 months) after surgery. Half of these recurrences occurred following wide excision. At most recent follow-up, 28 patients (23%) died of their disease.
*Conclusions:* The use of neoadjuvant chemotherapy given systemically or via ILP, attempted wide excision, and adjuvant radiation and chemotherapy achieve low recurrence rate in deep soft-tissue sarcomas of the extremities which are prone to high risk of recurrence.

**Follow-up of 33 patients with mega-endoprothesis**

O. Dubravko, S. Miroslav, K. Robert, O. Ivna[1]
*(Department of Orthopaedic Surgery, University of Zagreb, School of Medicine, and [1]Public Health CENTRE, Zagreb)*

Limb sparing surgery is a great challenge in contemporary oncology, because the reconstruction of the skeleton is performed in patients with the most aggressive bone tumors. From 1987 to 1999, at Department of Orthopaedic Surgery School of Medicine University of Zagreb, special modular uncemented mega-endoprostheses were implanted in 52 patients following '*en bloc*' resection. Indications were: active and aggressive benign tumors (eight patients, 15.4%); all types of malignant bone tumors (37 patients, 71.1%); tumor-like lesions (three patients, 5.8%); massive destruction of the proximal femur after severe war injuries or instability of standard hip endoprosthesis (four patients, 7.7%). Osteosarcoma was resected in 16 patients, Ewing sarcoma in eight, chondrosarcoma in six, metastasis in two, fibrosarcoma, synoviosarcoma, periosteal osteosarcoma, MFH and leiomyosarcoma in one patient, respectively. Among treated patients, 28 (53.8%) were females and 24 (46.2%) males. The youngest patient was an 11-year-old girl with osteosarcoma, and the oldest a 66-year-old patient with chondrosarcoma. Salvage surgery of the hip joint was performed in 25 (48.1%) and that of the knee joint in 27 (51.9%) patients. '*En bloc*' resection and salvage surgery of the knee joint for the destruction of the distal femoral region and that of the proximal tibia were done in 20 and seven patients, respectively. The median length of the proximal femoral resection was 17 cm; of the distal femur 16.4 cm; and of the proximal tibia 13 cm. The treatment results were analyzed in 33 patients after five or more year's of follow-up. Of these 33 patients. 13 (39.4%) had died, two (6.1%) disappeared because of the war situation, and 18 (54.5%) showed no evidence of disease at the last follow-up. Three of 18 patients have contracture of the knee joint, one patient has a fused knee after salvage surgery, and a girl with active hepatitis and salvage hip surgery is waiting for reoperation. The results of treatment obtained in our patients with bone resection surgery and the modular uncemented mega-endoprosthesis implantation showed a good outcome. One major concern regarding tumor resection and joint reconstruction with the special modular uncemented mega-endoprothesis is the high price of the mega-endoprothesis.

**Microsurgical treatment under intraoperative electrophysiological nerve monitoring for schwanoma excision**

F. Portabella, J. Casa as, M. Ordu a, J. Serra[1]
*(Orthopedics Department and [1]Neurology Department, Ciutat Sanitaria Bellvitge, Hospitalet Llobregat, Barcelona, Spain)*

*Methods:* Schawanomas are nerve tumors characterized by slow and solitary development. From 1996 to 1999, eight patients (four male, four female) were surgically treated for nerve tumors. Age ranges from 42 to 59 years old. Nerve tumors were localized in median nerve (two cases), posterior tibial nerve (three cases), ulnar nerve (one case) and brachial accessory nerve (two cases). Before surgery nerve function was evaluated: muscular testing was normal, some patients present dysesthesia in ther region of the nerve, and Tinel sign was positive on tumor localization. Nerve conduction studies was normal also. Diagnoses were done by IRM
*Results:* Microsurgical excision was performed under intraoperative electrophysiological nerve monitoring in order to evaluate fascicule damage during surgery. Clinical outcome presents Tinel sign in the lesion site, and dysesthesia and allodynia persist for a year. No tumor recurrences occur.
*Conclusions:* Although schwanomas are infrequent and clinical nerve affectation is poor, surgery must be careful because nerve damage occurs, so microsurgical technique and intraoperative electrophysiological nerve monitoring are important to evaluate nerve injury and nerve reconstruction.

**The use of polyethylenterephtalate Trevira tube to improve functional results in prosthetic reconstructive surgery after bone tumour resection in the proximal humerus: clinical, functional and radiographic considerations at 8 years follow-up**

M.A. Rosa, M. Galli, M. Tortora, G. Falcone & V. De Santis
*(Orthopaedic Department, Catholic University, Rome, Italy)*

*Study and aim:* Musculoskeletal tumour resection often requires the sacrifice of important anatomical structures, with a consequent functional deficit of the interested limb. There are many possibilities for reconstruction after a wide resection of the proximal humerus. The authors report their experience using megaprostheses (modular or custom-made). A new system for the reconstruction of soft tissues, to obtain a good and fast functional recovery of the involved limb, is also presented.
*Methods:* From January 1986 to 1999, 15 megaprostheses of the proximal humerus were implanted at the orthopaedic department of the Catholic University of Rome. All patients, seven males and eight females, were affected with primitive or secondary (metastases) malignant bone tumours with different histology. The preoperative planning was carried out using plain X-ray with a known magnification, to establish the length of the prosthesis. CT scan and MRI were useful to define the limits of the lesion and its extension. Soft tissue reconstruction, in eight cases, was performed using a mesh (Trevira tube), dressed all along the prosthesis. The patients were mobilized at the second post-operative day using an external device for passive mobilization, and at the seventh post-operative day they began active movements to strengthen the muscular masses and to recover joint function. The functional results were evaluated considering muscular strength, range of movement and residual pain, and they were divided into good, fair and poor.
*Results:* At a medium follow-up of 2 years, for the surviving patients, we have obtained a good functional recovery of the articular movements. We evaluated the results dividing the patient into two groups according to the level of bone resection (below or above deltoid insertion). There were better functional results in patients with resection above the insertion of the deltoid muscle. We have not observed complications related to the prosthesis. In only one case have we seen a probable intolerance to the Trevira tube.
*Conclusions:* The level of the resection is a main factor in order to obtain a good functional recovery. The articular function depends on the size of the resection. A wide resection (below deltoid insertion) with the sacrifice of large muscular masses and of the osteoarticular structures can represent a functional amputation. In our experience we observed that the use of modular prostheses in reconstructive orthopaedic surgery is advantageous because of its versatility, compared to other reconstructive systems. Moreover the use of the Trevira tube, for easy anchorage of the myotendinous structures, allows a quick functional recovery offering the patients considerable advantages for their social life.

**Uncemented conical femoral stem fixation in tumor TKA**

K.A. Siebenrock & F.T. Ballmer
*(Department of Orthopaedic Surgery, University of Berne, Switzerland)*

The principle of femoral stem fixation introduced by Wagner was adapted to total knee replacement for bone tumors. Femoral fixation was obtained by an uncemented conical titanium alloy stem with a coarse surface and eight sharp longitudinal ribs. Primary stability is achieved by the longitudinal ribs cutting into the cortical bone, and secondary stability has been evidenced by osseous ongrowth on the ribs. Preliminary experiences of this type of TKA implanted minimally 6 years ago in tumor patients are reported. The long conical femoral stem was connected to the original femoral component of a semiconstrained GSB total knee revision prosthesis (Sulzer Inc., CH) by a standard 12/14-mm cone. The tibial component was cemented in the usual manner. This type of prosthesis was used in three women and three men with a mean age of 36 years (7–75 years) over a 3-year time period. Distal femoral tumors included central osteosarcoma in four cases, recurrent paraosseal osteosarcoma and dedifferentiated chondrosarcoma each in one patient. Two patients died within 1 year after surgery due to lung metastases. The four surviving patients had a mean follow-up of 7.5 years (6–8.5 years) without evidence of tumor disease. Bone remodelling and a continuous solid bone–implant interface was observed in all cases without clinical or radiographic signs of femoral stem loosening. Complications included rotational instability of the cone attachment in two patients which required surgical correction. During the same surgery a loose cemented tibial compent was revised in one of these patients. This type of uncemented conical femoral stem fixation for TKA in tumor patients has maintained clinical and radiographic stability after a mean follow-up of 7.5 years. It seems promising and will be continuously used in our department. The cone system between the conical stem and the femoral prosthetic component has been modified in the meantime.

**The role of salvage operations in the treatment of the patients with the tumor of shoulder girdle**

N. Trapeznikov, M.V. Sokolovsky & A. Amiraslanov
*(N.N. Blokhin Cancer Research Centre, Kashirskoye sh.24, 115478, Moscow, Russia)*

The aim of the present study is an analysis of 75 patients with tumor of shoulder girdle bones from 1992 to 1998. The males represented 50.7% and the females 49.3% of patients. The age of 45.4% of patients was from 14 to 30 years. In 68 patients (90.7%) the tumor was localized in the humerus, and in seven (9.3%) in the scapula. By the histological structure, the patients were distributed into following groups: osteogenic sarcoma, eight patients; chondrosarcoma, 27 patients; parosteal sarcoma, six patients; giant cell tumors, 20 patients; MFH, four patients; benign tumors, 10 patients. The following operations were performed: resection of proximal part of humerus with endoprosthesis replacement, 22 patients (29.3%); interscapularis-pectoralis resection, 21 patients (28%); segmental resections with autoplastics, nine patients (12%); segmental resections with alloplastics, 11 patients (14.7%); segmental resections without plastics, six patients (8%); marginal resection, three patients (4%); excochelation tumor, three patients (4%). Relapses were observed in 17 patients: in 27.3% after endoprosthesis replacement; in 69.7% after segmental resections with auto- and alloplastics; in 19% after interscapularis-pectoralis resection. In eight (40%) of 20 patients treated by segmental resections with auto- and alloplastics, post-operative complications were observed: fracture and absorption of transplant. Thus, based on the conducted investigation, we conclude the salvage operations are indications for the treatment of shoulder girdle bone tumor.

**'Spontaneous regression' of pulmonary metastases in three patients with bone and soft tissue sarcomas**

S.J. Strauss, A. McTiernan & J.S. Whelan
*(The London Bone and Soft Tissue Service, The Middlesex Hospital, Mortimer Street, London W1N 8AA, UK)*

Spontaneous regression of cancer is a rare phenomenon and very few cases have been reported in bone or soft tissue sarcomas. Three cases were seen at a single centre between 1992 and 1999. Patient 1 presented with a retroperitoneal alveolar soft-part sarcoma. A staging CT scan demonstrated an isolated lesion in the right lung consistent with a pulmonary metastasis. Six months after surgical excision and radiotherapy to the primary tumour, the lung lesion became undetectable for 4 years. The patient then developed brain metastases. In patient 2, a CT scan demonstrated multiple pulmonary metastases 2 years after treatment for a localised synovial sarcoma. The patient declined treatment and 3 months later a CT scan showed a reduction in the size and number of nodules. Follow-up is continuing. Patient 3 developed multiple pulmonary metastases, demonstrated on CT scanning, 4 years after surgery for a local recurrence of a Ewing's sarcoma. Follow-up showed regression in a number of lesions and no progression in others over a 2-year period. The patient died of unrelated causes. Sarcomas are a heterogeneous group of tumours with variable natural histories but pulmonary metastases are invariably associated with poor outcome. Histological confirmation was not obtained in these patients, but radiological appearances are consistent with metastases. The mechanisms for spontaneous regression remain unclear, but the most cited cause is stimulation of host immunity by, for example, surgery or infection. Patient 1 underwent surgery. Alternative explanation is required for the other patients.

**External fixation (EF) in pathological fractures (PF) of long tubular bones**

V. Tepliakov, S. Tkachev, G. Matchak, M. Aliev & N. Trapeznikov
*(N.N. Blokhin Cancer Research Center, Kashirskoye sh. 24, Moscow 115478, Russia)*

*Purpose:* To evaluate the role of EF in the treatment of patients with PF.
*Patients and methods:* Between 1994 and 1999, the Ilizarov EF was used for fixation an reposition of PF in 22 patients with primary and metastatic tumours. There were 14F/8M, age 17–70 years. The distribution of cases according to histology was as follows: two patients with Ewing sarcoma, three with lymphosarcoma, two with osteosarcoma, one with fibrous osteodysplasy and one with malignant fibrous hystiocytoma, eight with metastatic breast cancer, three with metastatic renal cancer, one with melanoma and one with gastric cancer. The location of PF was neck of femur in nine cases, proximal femur in four, diaphysis femur in three, distal femur in three, neck of humerus in one and humerus diaphysis in two. EF was performed 3–30 days (median 11) after PF. Angular deformation with extremity length discrepancy from 2 to 8 cm was observed in 12 cases. The correction of PF was performed in 10 patients (six patients intraoperatively and four patients postoperatively).
*Results:* All patients were activated in 3–5 days after EF. Simultaneously, it was possible to administer local radiotherapy in 12 patients, in two of them with intra-arterial chemotherapy and seven with systemic chemotherapy. Six patients received hormone-, immuno- or chemotherapy alone and four received no treatment. The definitive PF consolidation was marked in 14 (64%) patients in periods from 173 to 372 days (average 171) after EF. After combined treatment with EF, three patients underwent delayed bone resections and reconstruction with endoprosthesis, allograft and vascularised autograft. At median follow-up of 21 months, the functional results were excellent in 10 (64%), good in four and satisfactory in three (14%). All patients have had acceptable life quality, irrespective of disease course. Five patients (23%) died from disease during the first year of follow-up.
*Conclusion:* EF permits to obtain a rigid fixation of PF, early activation of the patients and satisfactory conditions for combined treatment administration. In selected cases it is possible to perform delayed limb-salvage procedures.

**Clinical and radiological findings in classical chondrosarcoma with emphasis on a simple and reproducible grading system**

H. Welkerling[1], S. Kratz[2], G. Delling[3] & V. Ewerbeck[2]
*([1]Departement of Orthopaedic Surgery, University of Graz, [2]Departement of Orthopaedic Surgery, University of Heidelberg, and [3]Departement of Osteopathology University of Hamburg, Germany)*

*Study:* 35 chondrosarcomas (CS) and 16 enchondromas (EC) were analyzed in a retrospective study.
*Aim:* The purpose was to verify whether a correlation exists between the grading (Welkerling *et al.*, 1996), recurrence rate, and radiological and clinical findings.
*Methods:* 35 patients with CS and 16 patients with EC, both with complete documentation, were managed at the Department of Orthopaedic Surgery of Heidelberg University. The study included X-ray analysis, histological analysis, grading (based only on nuclear size and nuclear polymorphism) and evaluation of clinical data. EC were separated from low grade CS by growth pattern (Mirra, 1989).
*Results:* The mean age was 50 years; 6% of all lesions were located in the trunk and 46% in the long bones. The mean follow-up time was 40 months; 51% of the lesions were grade 1, 31% grade 2 and 17% grade 3; 91% of the patients with CS (88% of grade 1 CS) reported pain compared to only 44% with EC. Local recurrence was observed in 26%. Metastasis occurred only in one patient (grade 3 CS). All except one of the grade 3 tumours had local recurrence; 91% of the lesions were locally destructive, and in 57% an extraosseous component could be observed. No local recurrence was observed in Lodwick's grade I lesions. All of the grade 3 CS were classified Lodwick's grade II or III as well as 80% of the grade 2 CS; 85% of the CS and 82% of grade 1 lesions had blurred calcifications in comparison to only 19% of the EC cases. Cortical destruction could be observed only in 19% of EC, but in 94% of grade 1 CS. None of the EC lesions had an extraosseous component, whereas 47% of grade 1 CS did. Histologically there was no significant difference between the presence of double nuclei in EC (88%) and CS (94%). No difference of studied cytological features could be observed between grade 1 CS and EC.
*Conclusions:* The reproducible and simple grading system (Welkerling *et al.*, 1996) correlates with the frequency of local recurrence and radiological appearance. The radiological findings of grade 2 and 3 tumours were much more aggressive in comparison to grade 1 lesions. An extraosseous component could be found more frequently in grade 2 and 3 CS. EC and grade 1 CS are distinguishable by growth pattern, but not by cytological features. Cortical destruction could be observed more frequently in grade 1 CS and the presence of an extraosseous component and blurred calcifications are suspicious of malignancy in differentiation of EC and low grade CS.

**Internal hemipelvectomy—a less mutilating form of operational treatment of bone malignancies in children and youth**

W. Wozniak, M. Rychlowska-Pruszynska, M. Kuczabski, T. Izbicki, T. Walenta, A. Szafranski, K. Bilska, M. Lorkowska-Precht & J. Kijowski
*(Clinic of Oncological Surgery in Children, National Research Institute of Mother and Child, Kasprzaka str. 17 A, PL 01-211 Warsaw, Poland)*

In the period of 1985–1999 more than 250 patients with primary bone malignant tumours (osteosarcoma, Ewing sarcoma, chondrosarcoma) were treated in the National Institute of Mother and Child in Warsaw. In all of them contemporary methods of complex treatment were applied, including chemotherapy, surgery and/or radiotherapy. Localisation of primary neoplasm focus in the pelvis is less common and the radical surgery is problematic. We present a group of 13 patients in whom following operations were performed: two

'classical' hemipelvectomies (excision of pelvis bone with exarticulation of lower extremity); 11 internal hemipelvectomies (excision of pelvis bone sparing the lower extremity: two patients, type I, hip bone ala excision above the hip articulation; one patient, type II A, excision of the hip bone ala with the hip articulation; two patients, type II B, ischiadic and pubic bone excision with the hip articulation; four patients, type II C, excision of the hip bone ala with the hip articulation, pubic and ischiadic bones; two patients, type III, pubic and ischiadic bones excision under the hip articulation. Internal hemipelvectomies have been made mainly in the last 2 years. Ten patients are alive with a follow-up of 8–48 months. The patients have been rehabilitated with regard to the salvaged limb.
*Conclusions:* (1) The internal hemipelvectomy is a less mutilating operational treatment, performed within the complex therapy of malignant bone tumours in children and youth. (2) The internal hemipelvectomy allows the limb salvage and is efficacious with regard to patient's walking. (3) The internal hemipelvectomy requires a very well prepared operation team, good technical background allowing reconstruction of the resected bones and rehabilitation in the postoperational time.

### Nurses Symposium

### Protocol of pre- and post-operative nursing of patient treated by resection and prosthetic reconstruction of the knee for bone tumors

S. Urrai, P. Ruggeri & S. Mini
*(Istituto Ortopedico Rizzoli, Bologna, Italy)*

The aim of this paper was to define the protocol of pre-operative and post-operative nursing applied in the Surgical Department of Orthopedic Oncology at the Istituto Rizzoli for patients treated by knee resection for bone tumors and reconstruction with modular uncemented prosthesis (HMRS prosthesis). A series of 435 patients treated between 1982 and 1997 were reviewed, including 324 cases of resection of the distal femur, 106 of the proximal tibia and five of extrarticular resection of both distal femur and proximal tibia (removed '*en-bloc*'). There were 249 males and 186 females, whose age ranged from 5 to 68 years. Most frequent histological diagnoses were osteosarcoma (276 cases), chondrosarcoma (31 cases), Ewing's sarcoma (18 cases) and malignant fibrous histiocytoma (25 cases). Most of the lesions were staged II B. All our nursing forms were reviewed for all these patients. A remarkable change was observed over the years in nursing procedures for such patients, namely related with an increasing experience in the management of complications. Our nursing protocol is reported as defined at present and currently in use in our Surgical Department for these patients. This protocol is divided into the following phases: pre-operative preparation of the patients; immediate post-operative nursing of the patient (first 12 h); post-operative nursing of the patients and beginning of functional rehabilitation.

### The protocolization of nursing care as a measure to diminish complications in the use of 'implanted port'

P. Miqueleiz, C. Echarri, A. Belen Mu oz, S. Pérez & E. Marín
*(Paediatric Ward, University Clinic of Navarra, Pamplona, Navarra, Spain)*

Since the year 1983, the pediatric department of the CUN uses the 'implanted port' as a means of reaching the venous tract in children with onco-hematoligical diseases. A series of complications have been found derived from the use of this system in both the bibliography studied as well as through experience, principally obstructions and infections. A protocol of nursing activities was elaborated based on a comparative study to demonstrate that the complications can be diminished by means of its application. The comparative study of this protocol is presented with the purpose of demonstrating its effectiveness.

### Has chemotherapy in young patients with osteosarcoma any impact on weight?

C. Forni, L. Loro, T. Mazzei, C. Beghelli, M. Tremosini, A. Biolchini, P. Simoni, A. Treggiani, E. Dandolo, L. Loro & T. Coccia
*(Servizio di Chemioterapia degli Istituti Ortopedici Rizzoli, Bologna, Italy)*

The aim of this study was to evalutate in a period of 9 months how chemotherapic drugs affect weight in patients with osteosarcoma of the extremity treated with neoadjuvant chemotherapy (HD MTX, ADM, IFO, CDP). The weight of 237 patients was monitored at the beginning, during and at the end of therapy between January 1993 and November 1998. The response to antiemetic therapy was contemporarily monitored in order to evaluate a possible relationship with both weight loss and emesis. At the end of the therapy 60.8% of patients showed a weight increase of 10.9±7.2%, 33.3% a loss of 7.6±5.3% while with 5.9% of the patients no variation was recorded. The weight increase resulted proportional to both therapy duration ($p$=0.04) and number of cycles ($p$=0.003). No relation could be noticed between weight variation and characteristics of patient (sex, age, origin, lesion site, kind of performed protocol and incidence of emesis). In conclusion, we observed that opposite to the commonly accepted opinion that chemotherapy leads to weight loss with these patients and with this chemotherapic protocol, the majority of patients showed a weight increase. It is possible that these results can be referred to different factors such as cognitive distraction, autonomous kitchen, etc.

### Nursing process in order take care of children and teenagers affected by osteosarcoma

M.J. Lleo, M. Remiro, I. Irigoyen, D. Colunga & P. Lacasa

Malignant tumours of bone account for about 5.6% of the malignant tumours in children and teenagers, and they also represent 0.5% of all malignant tumours which affect humans. In the University Clinic of Navarra, osteosarcoma has been treated since 1972. The multidisciplinary treatment of this kind of disease with chemotherapy, surgery and RIO, has posed a challenge for nurses. Therefore, and basing this study on the acquired experience, we propose a new nursing process in order to take care of children and teenagers who are affected by osteosarcoma.

### The evaluation of a specialist teenage cancer unit using ethnography

S. Pearce, D. Kelly & A. Mulhall
*(UCL Hospitals NHS Trust)*

This presentation will illustrate the 'insiders' view of the culture of one of the few specialist adolescent oncology units in the UK. Cancer during the teenage years brings with it the challenges of cancer superimposed on the normal developmental transitions of adolescence. A growing literature points to the need for specialist expertise and services for teenagers and their families with cancer.

Despite this there is little research evidence to support the organisation of such services. The study used ethnography to evaluate a teenage cancer unit. This involved participant observation, the immersion of a researcher in the culture of the unit, together with in depth interviews of 10 parents, 10 teenagers and 16 members of the unit's interdisciplinary team. The study's collaborative and reflexive nature has been paramount. The field notes and the interview transcripts have been analysed to identify recurring themes. These include: the physical structure of the unit in terms of maintaining privacy, space and normality; the structure and rituals of the day; and the feelings of it being home. As one participant states '... it's almost like a second home ... unfortunately'. The language and the culture of cancer, the shared experience of being on the unit, together the individual experience of teenage cancer have also emerged as key themes from the data. This is the first study evaluating this specialist area in the UK. The findings will add to a sparse literature, stimulate the development of specialist practice and provide direction for the future organisation of adolescent oncology service provision.

**Nursing care in patients treated with internal hemipelvectomy for bone tumors**

S. Mini, S. Urrai, D. Donati & M. Mercuri
*(Orthopaedic Oncology Unit, Istituto Ortopedico Rizzoli, Bologna, Italy)*

In the Rizzoli Oncology Unit a number of surgical procedures for pelvic tumor are routinely applied. Internal hemipelvectomy and reconstruction due to malignant bone tumors are the most difficult to follow in terms of nursing care. Bed positioning, indwelling catheters (i.v., intra-cardiac, sondino nasogastric, bladder) and draining tube care, different therapy administration, requests for tests are only some of the main nursing activities. A retrospective study to measure the impact of nursing care in these patients has been addressed.

*Material:* From January 96 to December 99, 34 patients underwent internal hemipelvectomy with disruption of the pelvic ring. Ages ranged from 10 to 59 years (average 34). Fifteen patients were male and 19 female. The diagnosis was chondrosarcoma in 15 cases, osteosarcoma in 10, Ewing's sarcoma in five, GCT in two, leiomyosarcoma and synovial sarcoma in one each. The tumors were mainly located in the sacroiliac region in 11 cases (Group A), while the anterior arch was affected in another seven cases (Group B) and the hip area in the remaining 16 cases (four of which were located in the whole hemipelvis; Group C). Major reconstruction as allografts or prosthetic devices were applied in 22 cases, while the other 12 patients achieved no or minor reconstruction. The pre-operative check-up reported no significant data; however, in 22 patients a mean of six blood autoinfusion sacs (from one to 20) were collected to supply the estimate surgical blood loss.

*Results:* A number of parameters have been followed during the study. Some of which, such as fever control, blood tests (count, clot) indwelling catheter care, antibiotic, antipain medications, anti-thrombotic therapy, motility of the foot, dermal sensitivity and blood circulation control, and bed positioning were included in a protocol. The nursing care associated with internal hemipelvectomy patients resulted as one of the most time-consuming due to the magnitude of the operation and related needs. According to the type of operation the nursing activity can be more complex; the group of anterior pelvic arch resection showed longer hospital stay, more difficult following of evacuation, bladder catheter management and wound dressing, while in hip reconstruction the use of a spica cast is the rule. This study would be only a first step in reviewing the nursing care impact in different types of operations. More appropriate patient data collection forms will be necessary to correctly note all the patient care related activity and, hence, better evaluate the real complexity of the nursing occupation.
